# A Gain‐of‐Function Mutation in DELLA Selectively Alters Protein Interactions to Regulate Wheat Tiller Angle and Plant Height

**DOI:** 10.1111/pbi.70763

**Published:** 2026-10-08

**Authors:** Chaoqun Dong, Dejie Du, Yingying Li, Siyao Jia, Zhe Zhang, Yiqing Wang, Shuai Zheng, Xincheng Wang, Mingming Xin, Yingyin Yao, Lingling Chai, Zhongfu Ni, Qixin Sun, Jie Liu

**Affiliations:** ^1^ Frontiers Science Center for Molecular Design Breeding, State Key Laboratory of High‐Efficiency Production of Wheat‐Maize Double Cropping China Agricultural University Beijing China; ^2^ School of Food and Biological Engineering Beijing Vocational College of Agriculture Beijing China

**Keywords:** *Rht‐A1*, tiller angle, wheat

## Abstract

Plant architecture, especially plant height and tiller angle, plays a pivotal role in determining wheat yield. In this study, we characterize a wheat mutant termed *prostrate and semidwarf 1* (*prose1*), exhibiting both a prostrate growth habit and semidwarfism. Map‐based cloning of the causal gene of *prose1* identifies a semi‐dominant mutation in *Rht‐A1*–a homoeolog of the canonical Green Revolution genes–resulting in a glycine‐to‐glutamate substitution in the DELLA protein (designated *Rht‐A1h*). The mutant protein displays enhanced physical interactions with tiller angle regulators including TaPROG1, TaLAZY1, TaLAZY4 and TaD14, while showing weakened interaction with TaHST1L. Consistently, overexpression of *TaPROG1* phenocopies the enlarged tiller angle defect of *prose1*. We further demonstrate that Rht‐A1h and TaPROG1 together repress their co‐regulated downstream genes, exemplified by the shoot gravitropism‐regulating *TaWOX11*. Knockout of *TaWOX11* indeed recapitulates the enlarged tiller angle and defective shoot gravitropic response. A parallel study independently identified the same Rht‐A1h mutation and its interaction with TaPROG1; our work provides complementary mechanistic insight by establishing *TaWOX11* as a downstream target of this regulatory module. Together, our findings reveal a gain‐of‐function DELLA mutation that coordinates plant height and tiller angle through enhanced TaPROG1 interaction and *TaWOX11* repression, and offer a genetic resource for fine‐tuning wheat plant architecture in breeding.

## 1 Introduction

Wheat ( *Triticum aestivum* L.) is one of the most vital food crops worldwide, providing approximately 20% of the calories and 25% of the protein consumed by the global human population. It also serves as a significant source of essential trace elements. Enhancing wheat productivity is therefore critical for ensuring global food security (Dubcovsky and Dvorak 2007; Shiferaw et al. 2013). Plant architecture–a key agronomic trait that profoundly influences photosynthetic efficiency and grain yield in wheat–has long been a focal point in breeding programs. The ideotype concept, first introduced by Donald (Donald 1968), has motivated efforts to optimize plant form for enhanced productivity.

Among architectural traits, plant height and tiller angle are two pivotal components that significantly impact grain yield. Plant height is closely linked to lodging resistance; lodging can cause grain yield losses of 25%–50%, making height reduction a perennial objective in wheat breeding (Foulkes et al. 2011). The Green Revolution in the 1960s leveraged dwarfing alleles *Rht‐B1b* and *Rht‐D1b*, which confer gibberellin (GA) insensitivity through stabilized DELLA proteins, reducing stem elongation and significantly improving lodging resistance (Peng et al. 1999; Van De Velde et al. 2021). However, wheat is a hexaploid species harbouring three homoeologous *Rht‐1* genes: *Rht‐A1*, *Rht‐B1* and *Rht‐D1*. Unlike the well‐characterized *Rht‐B1b* and *Rht‐D1b*, the role of *Rht‐A1* remains largely unexplored, and no functional allele has yet been incorporated into modern breeding practice.

Tiller angle profoundly influences canopy architecture by affecting light penetration, spatial resource acquisition and microclimate conditions. Excessively wide angles may help reduce humidity‐related diseases but can also promote mutual shading and lower photosynthetic efficiency. Conversely, overly compact canopies with small tiller angle often suffer from inadequate light interception and higher susceptibility to pests and diseases, ultimately impairing yield potential (Wu et al. 2012; Yu et al. 2007; Zhang et al. 2018). An optimal tiller angle thus balances efficient light capture with disease resilience, enabling higher planting densities and improved yields (Zhang et al. 2018). Unravelling the genetic basis of both plant height and tiller angle is essential for designing improved plant architectures adapted to diverse environments.

Genetic mechanisms underlying tiller angle have been extensively studied in rice (Wang et al. 2022; Xu and Sun 2021; Zhang, Liu, et al. 2024; Zhang, Zhang, et al. 2024). Several major genes controlling tiller angle have been cloned, including *PROSTRATE GROWTH1* (*PROG1*), *Tiller Angle Control 1* (*TAC1*), *TAC4*, *LAZY1*, *LAZY4* and *TILLER INCLINED GROWTH 1* (*TIG1*) (Jin et al. 2008; Li et al. 2021, 2007; Tan et al. 2008; Wang et al. 2024; Yang et al. 2020; Yu et al. 2007; Zhang et al. 2019). These genes are often embedded in the gravitropic response pathway—a fundamental mechanism whereby plants perceive gravity and direct organ growth accordingly (Firn and Digby 1997; Volkmann and Baluška 2006). Stems exhibit negative gravitropism, growing opposite to the gravity vector, which helps reduce lodging and minimize yield loss (Chen et al. 1999). Molecular analyses reveal that the tiller angle controlling genes always function within complex networks; for instance, PROG1 and LAZY1 mutually repress each other to fine‐tune tiller angle and gravitropic response (Wang et al. 2023), while *TAC1* underlies differences between *indica* and *japonica* rice subspecies (Yu et al. 2007), and *TIG1* acts additively with other genes to promote cell elongation and tiller spreading (Zhang et al. 2019).

In wheat, research on tiller angle is still in its infancy, largely due to the complexity of its hexaploid genome, which harbours numerous repetitive sequences and homoeologous genes, complicating gene cloning and functional analysis. To date, only a few regulators have been identified. For instance, *TaHST1L* encodes a deacetylase that positively regulates tiller angle and number, likely through modulating auxin signalling and homeostasis (Zhao et al. 2023). Nevertheless, the overall genetic and mechanistic landscape of tiller angle control in wheat remains fragmented, warranting further investigation.

In this study, we characterized a novel wheat mutant, *prostrate and semidwarf 1* (*prose1*), which exhibits both prostrate growth and semidwarfism. Through map‐based cloning of the causal gene underlying the phenotypes of the *prose1* mutant, we identified a semi‐dominant mutant allele, *Rht‐A1h*, as the genetic basis of these phenotypes. The mutant protein carries a glycine‐to‐glutamate substitution that strengthens its physical interaction with TaPROG1, a key regulator of tiller angle. This enhanced Rht‐A1h–TaPROG1 complex amplifies repression of the shoot gravitropic regulator *TaWOX11*, resulting in disrupted shoot gravitropism and increased tiller angle. Our findings uncover a previously unrecognized mechanism by which *Rht‐A1* coordinately modulates plant height and tiller angle through enhanced protein–protein interactions. These findings establish *Rht‐A1* as a source of genetic variation for precisely tuning plant architecture–particularly tiller angle and provide a distinct breeding tool for designing wheat ideotypes.

## 2 Results

### 2.1 Phenotypic Characterization of *prose1*

Liangxing 99 (LX99) is an elite semi‐winter hexaploid wheat variety widely cultivated in the Huang‐Huai‐Hai winter wheat region of China. It carries the Green Revolution *Rht‐D1b* allele in the D subgenome and the wild‐type *Rht‐A1a* and *Rht‐B1a* alleles in the A and B subgenomes (genotype: *Rht‐A1a/Rht‐B1a/Rht‐D1b*), which contributes to its semi‐dwarf stature and lodging resistance, along with other elite loci that collectively underpin its excellent agronomic performance and broad adaptability. From an ethyl methanesulfonate (EMS)‐mutagenized population of LX99, we isolated a mutant line, designated *prostrate and semidwarf 1* (*prose1*), which exhibits reduced plant height and increased tiller angle.

At the seedling stage, the *prose1* mutant displayed a prostrate growth habit, with a significantly increased tiller angle compared to the wild‐type (WT) LX99 (Figure 1A). This prostrate growth habit results from impaired shoot gravitropism, leading to a looser plant architecture at the heading stage (Figure 1B). Furthermore, the degree of prostrate growth in *prose1* was highly sensitive to planting density, as the tiller angle increased significantly with decreasing planting density (Figure 1C, Figure S1).

**FIGURE 1:**
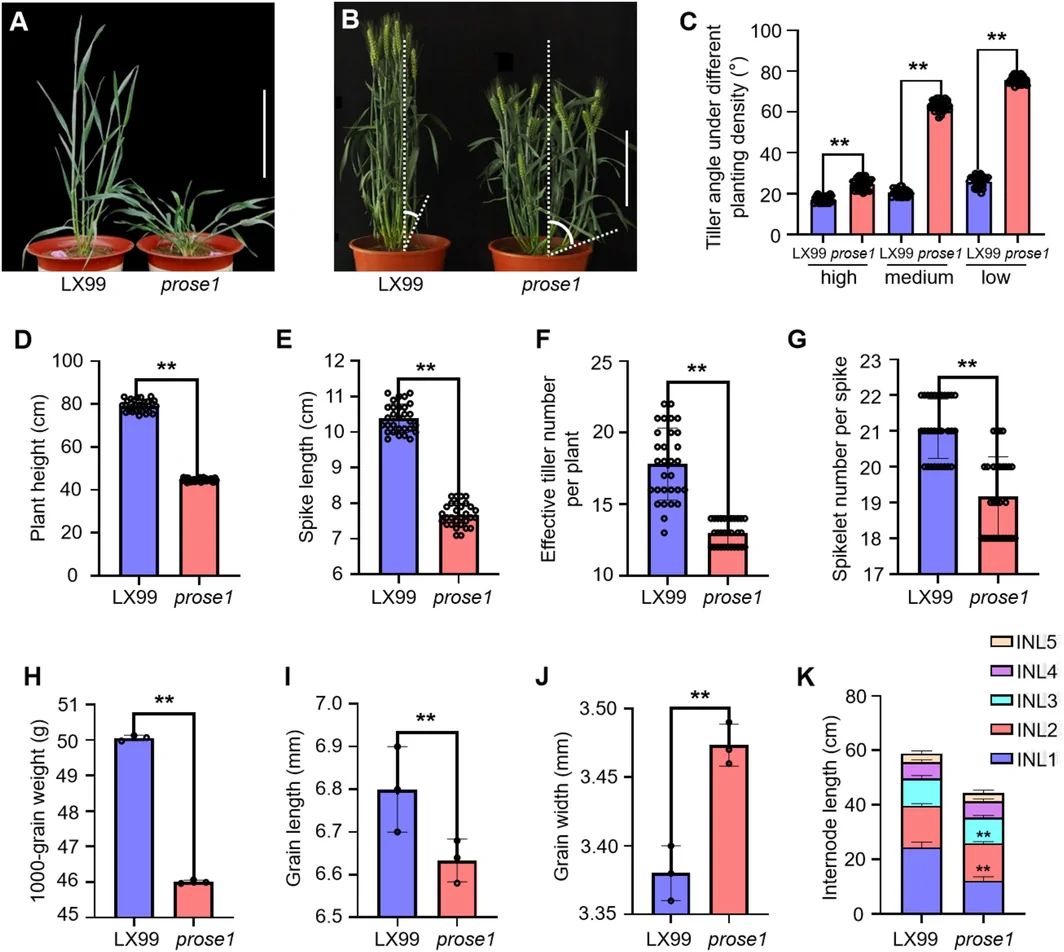
Phenotypic characterization of the prostrate and semidwarf mutant *prose1*. (A) The plant architecture of wild‐type Langxing99 (LX99) and *prose1* at the jointing stage under medium planting density (0.2‐m row spacing). Bar = 10 cm. (B) The plant architecture of LX99 and *prose1* at the grain filling stage under medium planting density (0.2‐m row spacing). Bar = 25 cm. (C) Tiller angles of LX99 and *prose1* under different planting densities at the jointing stage. High, 0.1‐m row space; medium, 0.2‐m row space; low, 0.3‐m row space. (D–G) Statistical analyses of agronomic trait comparisons between LX99 and *prose1*: Plant height (D), spike length (E), effective tiller number per plant (F) and spikelet number per spike (G). (H–J) Statistical analyses of grain traits between LX99 and *prose1* including thousand‐grain weight (H), grain length (I) and grain width (J). Measurements were based on three biological replicates. (K) Internode lengths (INL1–INL5 from top to bottom) of LX99 and *prose1* at grain maturity. Data in (C–K) represent mean ± SD; *n* = 30 individual plants (C–G, K) or *n* = 3 (H–J) biological replicates (**p < 0.01, Student's *t*‐test).

When compared to WT, *prose1* exhibited significantly shorter plant height and spike length, as well as lower effective tillers per plant, spikelet number per spike, grain length and thousand‐grain weight (Figure 1C–I). However, the grain width was significantly increased in *prose1* (Figure 1J). The reduced plant height of *prose1* was primarily due to the decreased lengths of the first (INL1, also known as peduncle) and second internodes (INL2) below the spike (Figure 1A,B,K), since they had significantly reduced cell length and cell diameter than those in WT (Figure 2A–F), consistent with observations in *Rht‐B1b* overexpression transgenic lines (Xu et al. 2023).

**FIGURE 2:**
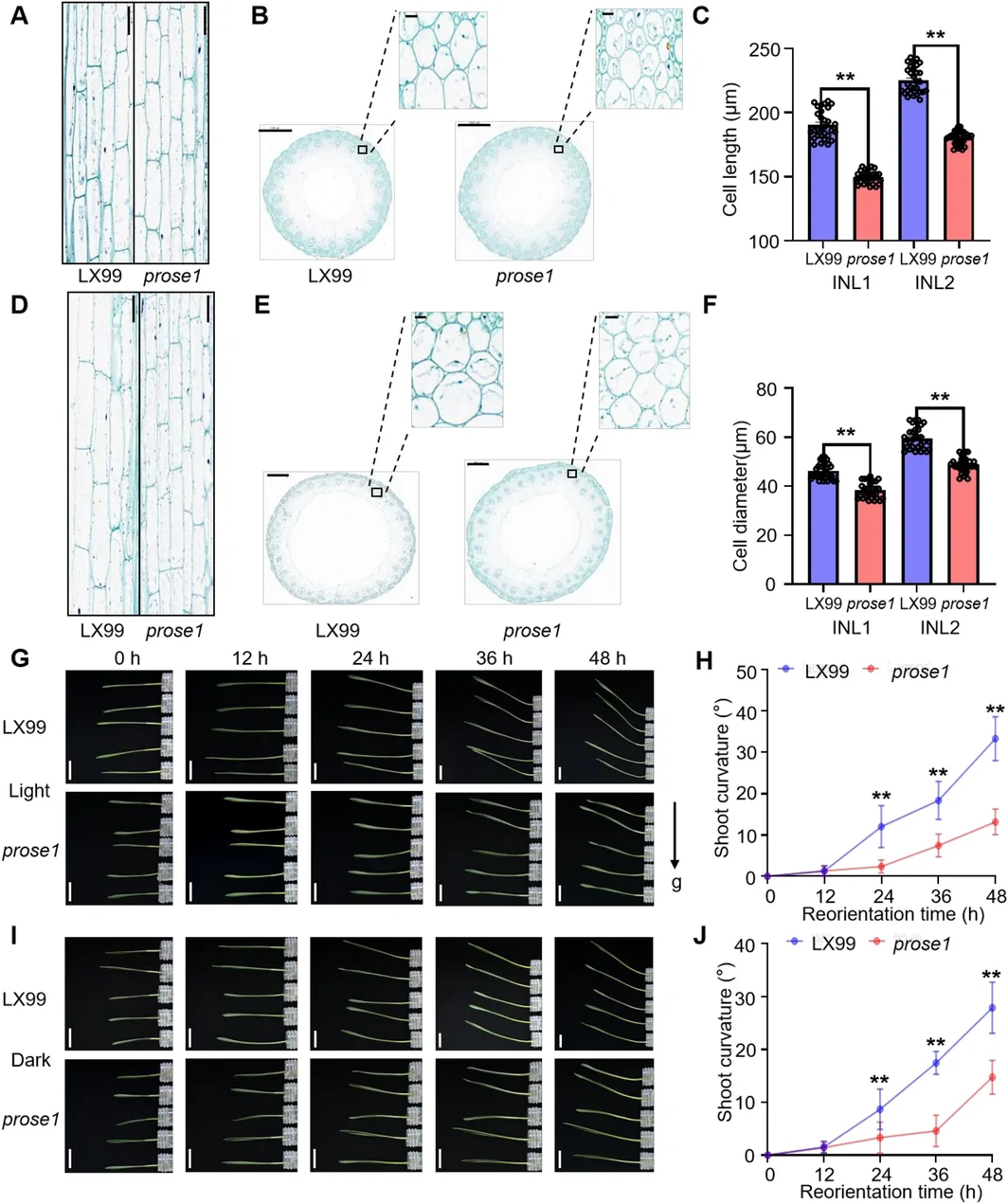
Histological analysis of internode cells and impaired shoot gravitropic response in *prose1* mutant. (A) Longitudinal sections of peduncle (INL1) cells. Bar = 50 μm. (B) Transverse sections of peduncle (INL1) cells. Complete section bar = 500 μm; enlarged view bar = 20 μm. (C) Comparison of cell length in INL1 and INL2. (D) Longitudinal sections of the first internode (INL2) cells. Bar = 50 μm. (E) Transverse sections of INL2 cells. Complete section bar = 500 μm; enlarged view bar = 20 μm. (F) Comparison of cell diameter in INL1 and INL2. (G) Shoot curvature of LX99 and *prose1* seedlings after 48 h of gravistimulation under light conditions. The arrow indicates the gravity vector (g). Scale bar = 2 cm. (H) Kinetics of shoot curvature under light conditions. (I) Shoot curvature after 48 h of gravistimulation in darkness. Scale bar = 2 cm. (J) Kinetics of shoot curvature under dark conditions. Data in (C) and (F) represent mean ± SD; *n* = 30 cells per genotype. Data in (H) and (J) represent mean ± SD; *n* = 30 individual plants (**p < 0.01, Student's *t*‐test).

Tiller angle is closely associated with shoot gravitropic response (Wang et al. 2022). To check whether the increased tiller angle of *prose1* was related to the same process, we examined the shoot gravitropic response of young *prose1* seedlings. The results showed that, when compared to WT, both light‐ and dark‐grown *prose1* seedlings displayed significantly impaired shoot curvature upon gravistimulation (Figure 2G–J), indicating that the shoot gravitropic response was partly compromised in *prose1*.

### 2.2 Map‐Based Cloning of the Candidate Gene for the *prose1* Phenotype

To elucidate the genetic basis of the *prose1* phenotype, we first crossed the mutant with its wild‐type progenitor, LX99. All F_1_ plants exhibited a semi‐prostrate and semi‐dwarf phenotype, indicating that the trait is controlled by a semi‐dominant allele. Following self‐pollination of the LX99 × *prose1* F_1_ plants, the resulting F_2_ population of 242 individuals exhibited segregation consistent with a single semi‐dominant gene model for tiller angle (homozygous *prose1* type: heterozygous: homozygous LX99 type = 1: 2: 1; *χ* ^2^ = 3.48 < *χ* ^2^_(0.05,2)_ = 5.99; Data S1), corroborating the findings from the F_1_ generation and further confirming that the *prose1* phenotype is determined by a single semi‐dominant gene.

To map the candidate gene for *prose1*, we performed bulked segregant RNA‐seq (BSR‐seq) analysis using extreme tiller angle bulks from the F_2_ population. SNP calling between the two bulks identified 19 high‐confidence SNPs (AFD > 0.8), of which eight were located on chromosome 4A (Figure S2A,B). The remaining SNPs were distributed on chromosomes 2A, 2B, 7A, 7B (two each) and 3B, 5B, 5D (one each) (Figure S2B). The enrichment of SNPs on chromosome 4A strongly suggests that the candidate gene resides on this chromosome.

For high‐resolution mapping, we generated an additional F_2_ population by crossing *prose1* with another elite hexaploid winter cultivar, Jing411 (J411) (genotype: *Rht‐A1a*/*Rht‐B1a*/*Rht‐D1a*). Analysis of this J411 × *prose1* F_2_ population, comprising 436 individuals, confirmed a segregation ratio consistent with a single semi‐dominant gene model (homozygous *prose1* type: heterozygous: homozygous J411 type = 1: 2: 1; *χ* ^2^ = 0.81 < *χ* ^2^_(0.05,2)_ = 5.99; Data S1). These results not only corroborated the conclusion that the *prose1* phenotype is governed by a single semi‐dominant gene but also ruled out potential effects from the known Green Revolution locus *Rht‐D1b* (which shows genetic differentiation between LX99/*prose1* and J411) in contributing to the prostrate phenotype observed in the *prose1* mutant, as our recent work revealed that the Green Revolution *Rht‐D1b* allele also contributes to the enlarged tiller angle phenotype (Zhang et al. 2025).

Genetic linkage analysis using 436 J411 × *prose1* F_2_ plants preliminarily mapped the candidate gene to a 20‐Mb region between markers *Indel‐5* and *Indel‐6*, a genetic distance of 4.7 cM. Using a larger J411 × *prose1* F_2:3_ population consisting of 1048 individual lines, we narrowed down the interval to a ~1 Mb region between markers *Indel‐10* and *Indel‐11* (Figure 3A). Based on the gene annotation of Chinese Spring IWGSC RefSeq v1.1 reference genome, this region contains seven high‐confidence genes: *TraesCS4A02G271000*, *TraesCS4A02G271100*, *TraesCS4A02G271200*, *TraesCS4A02G271300*, *TraesCS4A02G271400* and *TraesCS4A02G271500* and *TraesCS4A02G271600* (Figure 3A).

**FIGURE 3:**
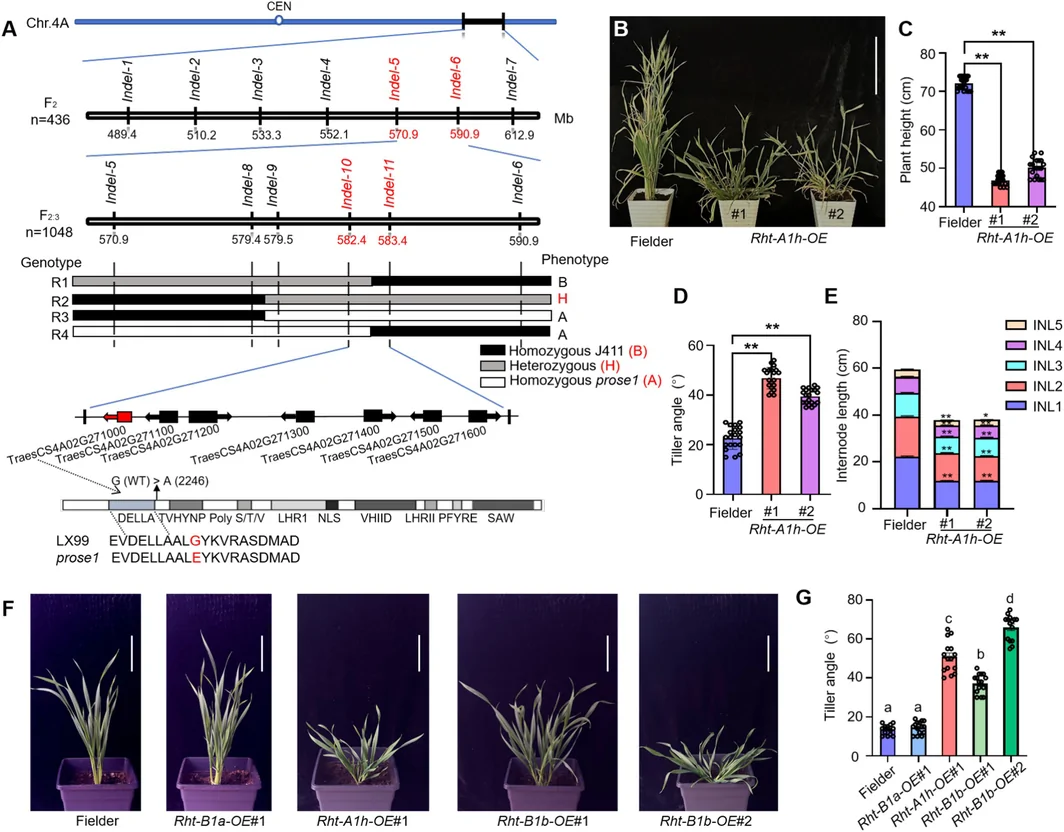
Map‐based cloning of *Rht‐A1* (*TraesCS4A02G271000*) mutation and phenotypic characterization of *Rht‐A1h* overexpression lines. (A) Genetic mapping of the *prose1* locus. The candidate region (∼1 Mb, in red) was fine‐mapped using F_2_ (*n* = 436) and F_2:3_ (*n* = 1048) populations. Genetic distances (cM) are indicated below markers. Gene annotation within the fine‐mapped interval. Arrows indicate transcriptional direction. Structure of TraesCS4A02G271000‐deduced Rht‐A1 protein, showing conserved DELLA domain. A G‐to‐A mutation in the coding sequence results in the amino acid substitution from glycine (G) in the wild type to glutamic acid (E) in the *prose1* mutant, occurring at the third residue downstream of the DELLA motif. (B) *Rht‐A1h*‐overexpressing lines (#1, #2) at the grain filling stage, showing increased tiller angle, reduced internode length and dwarfism compared to wild‐type Fielder. Scale bar = 30 cm. (C–E) Quantitative comparisons of plant height (C), tiller angle (D) and internode lengths (E) between Fielder and *Rht‐A1h‐OE* plants. (F) At the jointing stage, *Rht‐A1h*‐overexpression lines (#1) and *Rht‐B1b*‐overexpressing lines (#1, #2) exhibited significantly increased tiller angles compared to wild‐type Fielder. In contrast, *Rht‐B1a*‐overexpressing lines (#1) displayed erect growth habits similar to wild‐type Fielder. Scale bar = 10 cm. (G) Quantitative comparisons of tiller angle between Fielder, *Rht‐B1a*‐OE, *Rht‐A1h*‐OE and *Rht‐B1b*‐OE plants at the jointing stage. For (C–E), Data are mean ± SD; *p < 0.05, **p < 0.01 (Student's *t*‐test). For (G), data are mean ± SD. Significant differences were determined by one‐way ANOVA with Tukey's HSD post hoc test (*p* < 0.05). Different lowercase letters denote statistically significant differences between groups.

Sequencing analysis of these seven genes, including their coding and 2‐kb promoter regions, revealed only one G‐to‐A nucleotide variation in the coding region of *TraesCS4A02G271000* (Figure S3), which results in an amino acid substitution from glycine (G) to glutamic acid (E) in the *prose1* mutant compared to the WT. The mutation is located at the third amino acid downstream of the core DELLA motif (D‐E‐L‐L‐A), within the N‐terminal regulatory region of Rht‐A1. Specifically, the wild‐type sequence DELLAALG is changed to DELLAALE in the mutant, with the glycine (G) to glutamic acid (E) substitution occurring at the third residue after the DELLA motif. Given that *TraesCS4A02G271000* (A subgenome) is homoeologous to the Green Revolution genes *Rht‐B1b* and *Rht‐D1b*. In addition to the wild type Rht‐A1a, to date, there are six *Rht‐A1* allelic variations (*Rht‐A1b–g*) have been reported (Li et al. 2013), in this study, we designated the *prose1*‐derived *Rht‐A1* mutant allele as *Rht‐A1h*. We therefore consider *Rht‐A1h* as the most promising candidate gene for the *prose1* phenotype and have selected it for further functional analysis.

### 2.3 *Rht‐A1h* Overexpression Alters Tiller Angle and Reduces Plant Height

To validate the biological function of *Rht‐A1h*, we introduced a *Ubi::Rht‐A1h* construct (driven by the maize *Ubiquitin* promoter) into wheat cultivar Fielder (genotype: *Rht‐A1a/Rht‐B1b/Rht‐D1a*) to generate *Rht‐A1h‐OE* transgenic lines. Compared with the wild‐type, *Rht‐A1h‐OE* exhibited significant reductions in plant height and the lengths of each internode, leading to pronounced plant dwarfing. More importantly, *Rht‐A1h‐OE* also showed significantly increased tiller angle (Figure 3B–E), mimicking the prostrate habit of *prose1*. These phenotypic alterations were especially evident at the jointing stage (Figure S4).

In parallel, we generated *Rht‐B1a‐* and *Rht‐B1b‐*overexpression transgenic lines (*Rht‐B1a‐OE* and *Rht‐B1b‐OE*). Phenotypic analysis revealed that *Rht‐B1a‐OE* showed no significant difference in tiller angle compared to wild‐type plants, whereas *Rht‐B1b‐OE* displayed significantly enlarged tiller angles (Figure 3F,G), similar to those observed in *prose1* and *Rht‐A1h‐OE* lines. To further assess whether overexpression of the wild‐type *Rht‐A1a* allele affects plant architecture, we also generated *Rht‐A1a‐OE* transgenic lines. At the jointing stage, *Rht‐A1a‐OE* T_1_ plants exhibited tiller angles and overall plant architecture comparable to wild‐type Fielder (Figure S5A,B).

We examined the transcript levels of *Rht‐B1* in the *Rht‐B1a‐OE* and *Rht‐B1b‐OE* lines using qRT‐PCR with primers that amplify both *Rht‐B1a* and *Rht‐B1b* transcripts. Among the *Rht‐B1b‐OE* lines, line #1 showed approximately 4‐fold overexpression with moderate tiller angle enlargement, while line #2 showed approximately 12‐fold overexpression with more pronounced tiller angle enlargement (Figure S5C), indicating a positive correlation between *Rht‐B1b* expression level and the severity of the tiller angle phenotype. These results further support that the mutant alleles, rather than overexpression of the wild‐type proteins, are responsible for the enlarged tiller angle phenotype.

We next asked whether the single nucleotide mutation in *Rht‐A1* (*Rht‐A1h* vs. *Rht‐A1a*) is critical for the *Rht‐A1h*‐triggered repression of gravitropism, similar to the difference observed between *Rht‐B1b* and *Rht‐B1a*. To test this, we employed a well‐established modified carbon dot (MCD)‐mediated seed transient transformation strategy (She et al. 2025). This technique enables direct transient gene transformation in wheat seeds, allowing for sustained protein expression in developing seedlings—a system that closely mimics stable genetic transformation without requiring tissue culture or plant regeneration. Using this platform, we introduced *Rht‐A1a‐GFP* and *Rht‐A1h‐GFP* into Fielder seeds and monitored phenotypes in the resulting seedlings (Figure 4A–E). Seedlings expressing *Rht‐A1a‐GFP* exhibited normal shoot gravitropic responses indistinguishable from wild‐type Fielder controls. In contrast, seedlings expressing *Rht‐A1h‐GFP* recapitulated the impaired shoot gravitropic phenotype observed in the original *prose1* mutant (Figure 4A–D). Importantly, immunoblot analysis confirmed that the two constructs produced comparable protein levels (Figure 4E), confirming that the phenotypic difference is attributable to the Gly‐to‐Glu amino acid variation between Rht‐A1a and Rht‐A1h proteins. These results support that the mutated *Rht‐A1h*, rather than the wild‐type *Rht‐A1a*, is critical for impaired shoot gravitropism in wheat.

**FIGURE 4:**
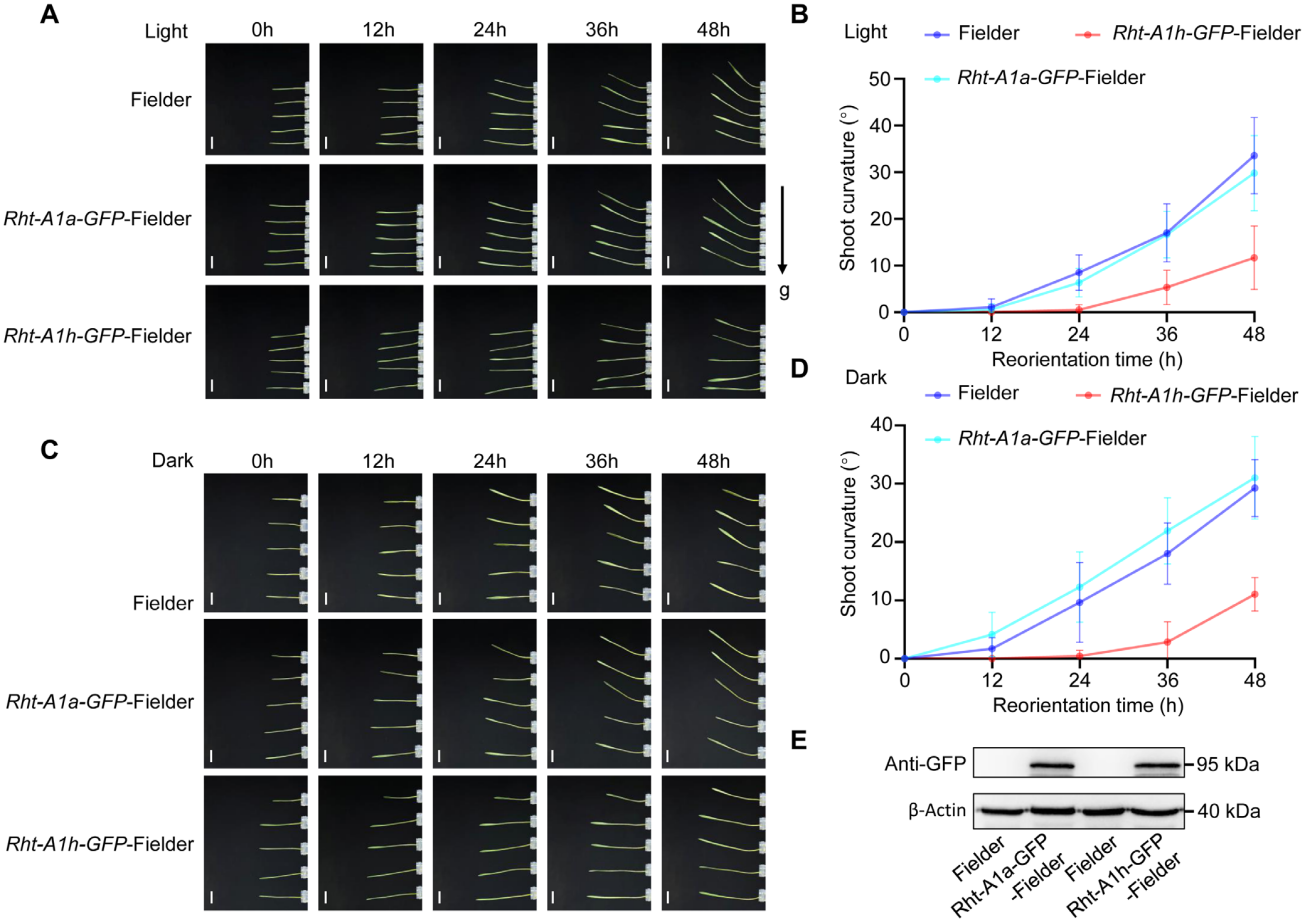
Transient expression of *Rht‐A1h*, but not *Rht‐A1a*, impairs shoot gravitropism in wheat. (A) Shoot curvature of Fielder, *Rht‐A1a‐GFP*‐Fielder and *Rht‐A1h‐GFP*‐Fielder seedlings after 48 h of gravistimulation under light conditions. Scale bar = 2 cm. (B) Kinetics of shoot curvature under light conditions. Data are mean ± SD; *n* = 30 individual plants. (C) Shoot curvature of Fielder, *Rht‐A1a‐GFP*‐Fielder and *Rht‐A1h‐GFP*‐Fielder seedlings after 48 h of gravistimulation under dark conditions. Scale bar = 2 cm. (D) Kinetics of shoot curvature under dark conditions. Data are mean ± SD; *n* = 30 individual plants. (E) Western blot analysis of total protein extracts from wild‐type Fielder and Fielder seedlings transiently expressing Rht‐A1a‐GFP or Rht‐A1h‐GFP, using Anti‐GFP antibody. β‐Actin served as the loading control.

We also employed *Rht‐B1a‐OE* and *Rht‐B1b‐OE* lines as controls in shoot gravitropic response assays. Our results showed that only *Rht‐B1b‐OE* lines—not *Rht‐B1a‐OE* plants—exhibited significantly impaired shoot gravitropic responses relative to wild‐type Fielder seedlings (Figure S6), consistent with our previous observations that only *Rht‐B1b‐OE* lines conferred obviously enlarged tiller angles (Figure 3F,G). Together, these results support that: (1) *Rht‐A1h* is the causal mutation for the prostrate phenotype of *prose1* and (2) the semi‐dominant mutations in *Rht‐A1h* and *Rht‐B1b*, but not the overexpression of the wild‐type RHT‐1 proteins, result in altered tiller angle.

Quantitative real‐time PCR (qRT‐PCR) revealed similar expression patterns for *Rht‐A1*, *Rht‐B1* and *Rht‐D1* across the tissues examined (Figure S7A). To further compare tissue‐specific expression patterns of *Rht‐A1* and *Rht‐B1*, we also introduced the *RUBY* reporter system (He et al. 2020) and generated constructs *pRht‐A1::RUBY* and *pRht‐B1::RUBY* driven by the native promoters of *Rht‐A1* and *Rht‐B1*, respectively, and introduced them into Fielder. Both genes were highly expressed in tiller buds at the jointing stage (Figure S7B), consistent with their role in tiller angle regulation. After heading, *Rht‐B1* expression was moderately higher than *Rht‐A1* in culms and spike axes, although the difference was not pronounced (Figure S7C–E). During grain filling, both genes showed similarly high expression in grains (Figure S7F).

In summary, genetic evidence confirmed that *Rht‐A1h* is the causal gene underlying the *prose1* phenotype. Notably, *Rht‐A1* and *Rht‐B1* exhibit broadly similar expression patterns across the tested tissues, and overexpression of both *Rht‐A1h* and *Rht‐B1b* produced a similar semi‐dwarfing phenotype and increased tiller angle. These findings, together with our recently published work supporting a role for *Rht‐D1b* in regulating tiller angle (Zhang et al. 2025), suggest that the mutant alleles *Rht‐A1h*, *Rht‐B1b* and *Rht‐D1b* exert similar effects in triggering the prostrate phenotype.

### 2.4 Rht‐A1h Physically Interacts With Multiple Tiller Angle Regulators Including TaPROG1

Given the role of Rht‐A1h in regulating tiller angle, we investigated its potential functional association with known regulators of tiller or spreading growth habit. Using a split‐luciferase complementation imaging (LCI) assay, we tested the interaction between Rht‐A1h and several key proteins involved in tiller angle control, including TaLAZY1, TaLAZY4, TaHST1‐like (TaHST1L), TaPROG1 and TaD14 (Figure 5A; Figure S8A,B). Strong interaction signals were observed for all these proteins when co‐expressed with Rht‐A1a. Notably, most of them exhibited stronger interaction with the mutant Rht‐A1h compared to wild‐type Rht‐A1a, whereas TaHST1L displayed the opposite pattern, showing preferential interaction with Rht‐A1a (Figure S8B). We extended this analysis to Rht‐B1 and found that Rht‐B1b, like Rht‐A1h, exhibited significantly stronger interaction with TaPROG1 than did Rht‐B1a (Figure S8C). To gain structural insight into this altered binding, we performed AlphaFold‐based protein structure predictions. The modelling revealed that the Gly‐to‐Glu substitution in Rht‐A1h induces a conformational change that may facilitate more efficient binding to interacting partners such as TaPROG1 (Figure S9A,B).

**FIGURE 5:**
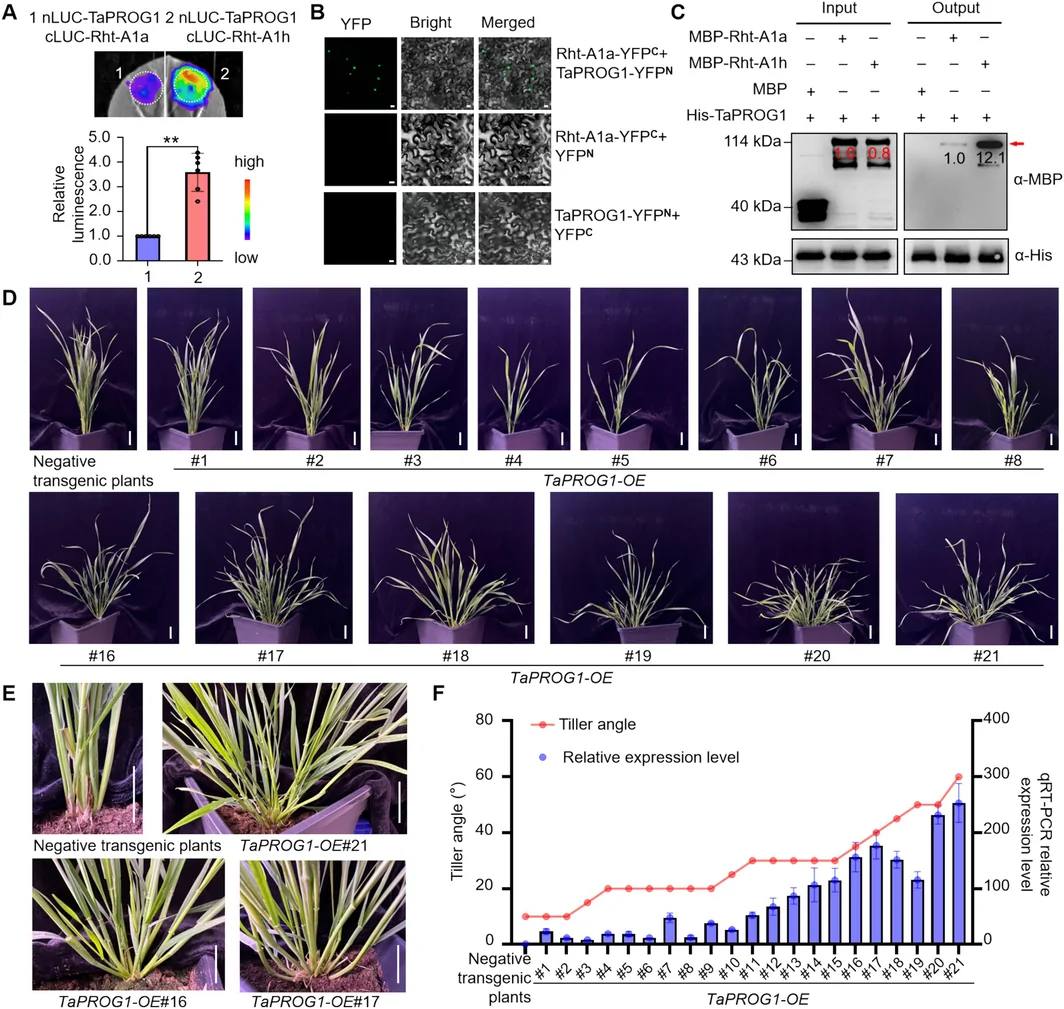
Rht‐A1 functionally associates with tiller angle regulator TaPROG1, with the Rht‐A1h mutation enhancing their interaction and *TaPROG1* overexpression increasing tiller angle. (A) Split‐luciferase complementation assays (LCI) detecting stronger Rht‐A1h–TaPROG1 interaction compared to Rht‐A1a–TaPROG1. (**p < 0.01, Student's *t*‐test). (B) Bimolecular fluorescence complementation (BiFC) confirms Rht‐A1a–TaPROG1 interaction in plant cells. Scale bar = 20 μm. (C) Pull‐down assays validate enhanced in vitro association of Rht‐A1h with TaPROG1 relative to the wild‐type Rht‐A1a. Red arrows indicate the target bands. (D, E) At the jointing stage, *TaPROG1*‐overexpressing lines exhibited significantly increased tiller angles compared to negative transgenic Fielder. Scale bar = 4 cm. (F) Quantitative real‐time PCR analysis of *TaPROG1* (tiller bases) transcript levels (blue bars, right Y‐axis) and tiller angle measurements (red dots with lines, left Y‐axis) in independent transgenic lines overexpressing *TaPROG1* shown in (D). *TaPROG1* expression levels were normalized to the endogenous reference gene and are shown relative to negative transgenic Fielder (set as 1). The results demonstrate a clear positive correlation between *TaPROG1* expression level and tiller angle: Lines with higher *TaPROG1* transcript abundance exhibit progressively enlarged tiller angles. Data represent mean ± SD from three technical replicates for expression analysis.

Among these candidates, TaPROG1—a well‐documented regulator of tiller angle in cereals (Jin et al. 2008; Tan et al. 2008)—showed the most pronounced difference in interaction strength between Rht‐A1a and Rht‐A1h, prompting us to prioritize it as a candidate for further investigation (Figure 5A; Figure S8A,B). The interaction between Rht‐A1a and TaPROG1 was confirmed to occur in the nucleus using bimolecular fluorescence complementation (BiFC) assays (Figure 5B). LCI assays revealed significantly stronger interaction for Rht‐A1h–TaPROG1 compared to Rht‐A1a–TaPROG1 (Figure 5A), which was further supported by in vitro pull‐down assays, in which MBP‐tagged Rht‐A1h pulled down more His‐TaPROG1 protein than MBP‐tagged Rht‐A1a (Figure 5C). Together, these results demonstrate that Rht‐A1 directly interacts with TaPROG1 both in vivo and in vitro and that the Gly‐to‐Glu substitution in Rht‐A1h strengthens this interaction.

To further assess the functional relevance of TaPROG1 in tiller angle regulation in wheat, we generated *TaPROG1*‐overexpression transgenic plants using the Fielder background. A total of 21 independent positive transgenic events were obtained, and qRT‐PCR analysis revealed that these lines expressed *TaPROG1* at levels approximately 8‐ to 253‐fold higher than those in negative transgenic plants (regenerated T_0_ lines without T‐DNA insertion) (Figure 5F). Phenotypic evaluation demonstrated a clear positive correlation between *TaPROG1* expression level and tiller angle: lines with higher *TaPROG1* expression exhibited progressively enlarged tiller angles (Figure 5D–F; Figure S10A). This dose‐dependent effect of *TaPROG1* in controlling wheat plant architecture provides genetic evidence that *TaPROG1* acts as a positive regulator of tiller angle in wheat. We next compared the phenotypes of *Rht‐A1a‐OE*, *TaPROG1‐OE* and *Rht‐A1h‐OE* transgenic lines at the jointing stage (Figure S10B). *Rht‐A1a‐OE* plants displayed tiller angles comparable to wild‐type Fielder, whereas both *TaPROG1‐OE* and *Rht‐A1h‐OE* lines exhibited significantly enlarged tiller angles.

Collectively, our results support a physical interaction between Rht‐A1 and TaPROG1, a tiller angle facilitator in wheat and demonstrate that this interaction is enhanced by the Rht‐A1h mutation.

### 2.5 Transcriptome Profiling Identifies Genes Differentially Expressed in *Rht‐A1h‐OE* Plants

To elucidate the downstream signalling associated with Rht‐A1h function in tiller angle regulation, we conducted transcriptome profiling using tiller bases collected from wild‐type Fielder and *Rht‐A1h‐OE* transgenic plants at the jointing stage. A total of 2042 genes were significantly upregulated and 1560 were downregulated in *Rht‐A1h‐OE* lines compared to the wild‐type (Figure 6A; Data S2). Gene Ontology (GO) enrichment analysis indicated that upregulated differentially expressed genes (DEGs) were associated with terms such as ‘auxin‐activated signalling pathway’, ‘response to jasmonic acid’, ‘response to salicylic acid’, ‘response to light stimulus’ and ‘response to auxin’ (Figure 6B; Data S3). In contrast, downregulated DEGs were enriched in ‘peroxidase activity’, ‘regulation of growth’, ‘ethylene‐activated signalling pathway’, ‘gravitropism’, ‘response to UV’ and ‘response to ethylene’ (Figure 6B; Data S4).

**FIGURE 6:**
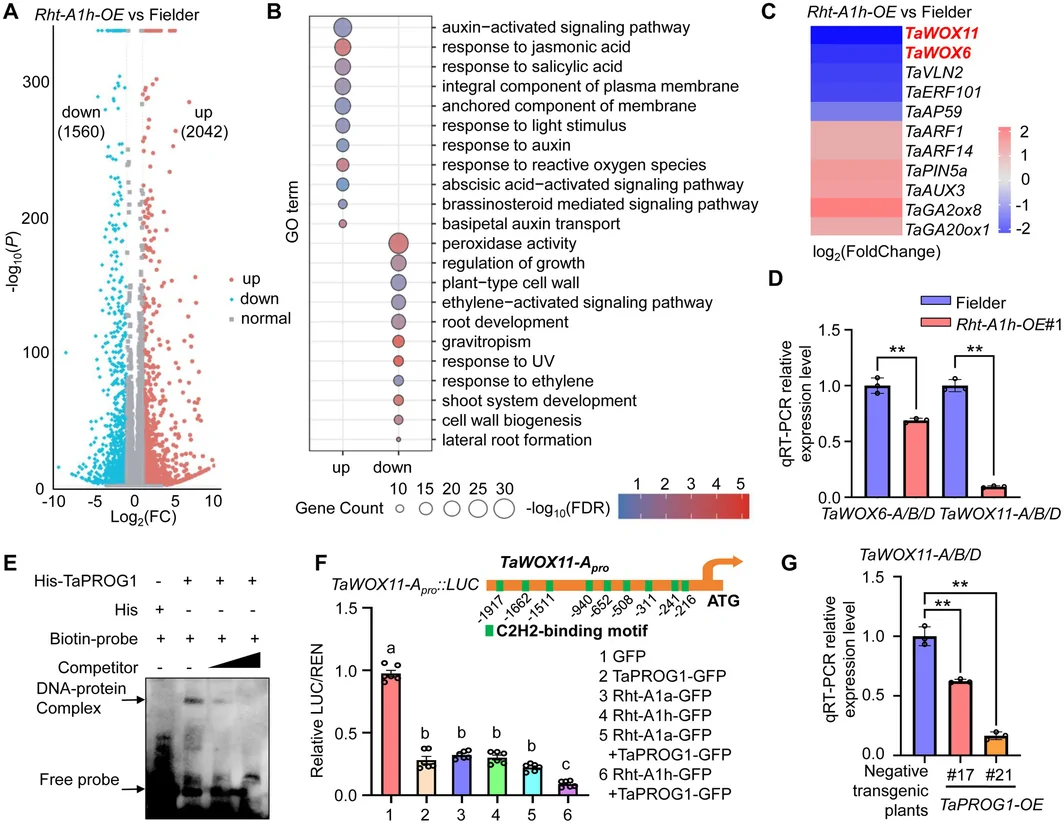
Transcriptome profiling and biochemical analyses support the transcriptional regulation of *TaWOX11* by Rht‐A1h and TaPROG1, with in vivo validation in *TaPROG1*‐overexpressing lines. (A) Identification of DEGs between *Rht‐A1h‐OE* and Fielder. (B) Gene ontology (GO) enrichment analysis of regulated genes showed distinct functional categories. (C) Heatmap analysis of selected hormone‐related genes reveals consistent expression changes in *Rht‐A1h‐OE*. (D) qRT‐PCR validation confirming the expression levels of *TaWOX6‐A/B/D* and *TaWOX11‐A/B/D* in *Rht‐A1h‐OE* (line #1) (*n* = 3 biological replicates). (E) EMSA analysis confirms the binding of TaPROG1 to the *TaWOX11‐A* promoter region. Competition assays with unlabeled probes show dose‐dependent reduction of TaPROG1‐DNA complex formation, demonstrating binding specificity. (F) Dual‐luciferase assay showing that TaPROG1 and Rht‐A1 independently suppress *TaWOX11* promoter activity, with additive effects when TaPROG1 and Rht‐A1h co‐expressed but not Rht‐A1a (*n* = 6 biological replicates). (G) qRT‐PCR analysis of *TaWOX11* expression in *TaPROG1*‐overexpressing lines (tiller bases). (*n* = 3 biological replicates). For (D), (F) and (G), data are mean ± SD. In (D) and (G), **p < 0.01 (Student's *t*‐test). In (F), significant differences were determined by one‐way ANOVA with Tukey's HSD post hoc test (*p* < 0.05) and different lowercase letters denote statistically significant differences between groups.

Notably, several key regulators of plant development were suppressed in *Rht‐A1h‐OE* plants. These include the *VILLIN2* (*VLN2*) gene, which is involved in polar auxin transport (Wu et al. 2015); *WOX11* and *WOX6*, which play roles in shaping shoot and root meristems (Zhang et al. 2018); and the APETALA2/ethylene responsive factor (AP2/ERF) family genes *ERF101* and *AP59*, which function in hormone signal transduction and plant growth/development regulation (Oh et al. 2009; Yoshihisa et al. 2022). Conversely, genes associated with auxin response and gibberellin metabolism were significantly upregulated. These include auxin response factor genes *ARF1* and *ARF14* (Gu et al. 2023; Zhang et al. 2026), the Aux/IAA gene *AUX3* involved in regulating auxin‐responsive signalling pathways (Qiao et al. 2021), *PIN‐FORMED5a* (*PIN5a*) encoding an auxin efflux carrier protein (Wang et al. 2009), as well as *GA2ox8* and *GA20ox1* (Guo et al. 2025; Zhang, Zhang, et al. 2024), which are involved in GA biosynthesis (Figure 6C). These results suggest that overexpression of *Rht‐A1h* disrupts the homeostasis and signalling transduction of multiple phytohormones, including auxin, ethylene and gibberellin, thereby potentially contributing to the observed alterations in tiller angle and plant architecture.

### 2.6 *TaWOX11* Is a Key Downstream Target Co‐Repressed by Rht‐A1h and TaPROG1

Among the DEGs, *TaWOX11* and *TaWOX6* were strongly repressed by *Rht‐A1h* overexpression (Figure 6C,D), suggesting that these *TaWOX* genes may be directly or indirectly targeted by Rht‐A1h proteins for transcriptional repression. To validate the relevance of this regulation in the native mutant background, we examined *TaWOX11‐A/B/D* expression in the original *prose1* mutant. qRT‐PCR analysis revealed that *TaWOX11‐A/B/D* transcript levels in tiller bases were significantly lower in *prose1* than in wild‐type LX99 (Figure S11B), consistent with the repression observed in *Rht‐A1h‐OE* lines. Furthermore, we predicted multiple C2H2‐binding motifs within the promoters of *TaWOX* genes (Figure 6F; Figure S11A), raising the possibility that TaPROG1—a C2H2‐type transcription factor—may directly bind to these promoters to regulate *TaWOX* transcription. This would position *TaWOX* genes as ideal downstream candidates to exemplify the biological relevance of the genetic and biochemical interaction between Rht‐A1h and TaPROG1.

To test this, we first conducted an electrophoretic mobility shift assay (EMSA), which confirmed that TaPROG1 directly binds to the *TaWOX11‐A* promoter region (Figure 6E). We next performed a transient transcriptional activation assay in *Nicotiana benthamiana* leaves using a luciferase (LUC) reporter driven by the native *TaWOX11‐A* promoter. Expression of TaPROG1‐GFP strongly suppressed LUC activity compared to the GFP control (Figure 6F), indicating that TaPROG1 not only binds to the *TaWOX11‐A* promoter but also represses its transcription. Similarly, Rht‐A1h‐GFP expression significantly reduced LUC activity, consistent with the observed transcriptional downregulation of *TaWOX11* in *Rht‐A1h‐OE* plants (Figure 6C,D).

Given the physical interaction between Rht‐A1a/Rht‐A1h and TaPROG1 (Figure 5A,C), we tested whether these proteins act synergistically to regulate *TaWOX11*. Notably, co‐expression of Rht‐A1h and TaPROG1—but not Rht‐A1a and TaPROG1—resulted in an additive repressive effect on *TaWOX11‐A* promoter activity (Figure 6F). This finding aligns with the stronger interaction observed between Rht‐A1h and TaPROG1 relative to Rht‐A1a and TaPROG1 (Figure 5A,C). We next tested whether Rht‐B1b, like Rht‐A1h, also regulates *TaWOX11* expression. Transient co‐expression assays revealed that Rht‐B1b and TaPROG1 synergistically repress *TaWOX11‐A* promoter activity (Figure S11D).

We further examined *TaWOX11‐A/B/D* expression in *TaPROG1‐OE* lines by qRT‐PCR and found that *TaWOX11‐A/B/D* transcript levels were significantly reduced upon *TaPROG1* overexpression (Figure 6G). To determine whether this repression affects all three *TaWOX11* homoeologs, we designed homoeolog‐specific primers for *TaWOX11‐A*, *‐B* and *‐D* and re‐examined their expression in *Rht‐A1h‐OE* and *TaPROG1‐OE* lines. The results showed that all three homoeologs were significantly downregulated in both backgrounds (Figure S11C), indicating that the regulatory effect is global rather than restricted to a single homoeolog. To further determine whether TaPROG1 directly binds to the *TaWOX11‐B* and *TaWOX11‐D* promoters, we performed EMSA assays using homoeolog‐specific probes. The results showed that TaPROG1 binds to all three promoters (Figure S11E,F), indicating that the regulation of *TaWOX11* by TaPROG1 is conserved across the A, B and D subgenomes.

Taken together, our results demonstrate that Rht‐A1h and TaPROG1 act synergistically to repress their common downstream target, *TaWOX11*.

### 2.7 Knockout of *TaWOX11* in Wheat Enhances Tiller Angle

To determine the biological significance of *TaWOX11* in regulating wheat tiller angle, we generated *Tawox11* null mutants (#1 and #2) via CRISPR/Cas9‐mediated editing, in which all three homoeologous copies from the A, B and D subgenomes were disrupted (Figure S12). Phenotypic analysis revealed that *Tawox11* mutants exhibited reduced plant height and, more importantly, significantly increased tiller angles (Figure 7A–D; Figure S13). These results support the conclusion that *TaWOX11*, as a downstream gene co‐regulated by Rht‐A1h and TaPROG1, plays a critical role in wheat tiller angle control. The prostrate growth habit of *Tawox11* mutants phenocopies that of *Rht‐A1h‐OE* plants, further reinforcing the functional link between these regulators.

**FIGURE 7:**
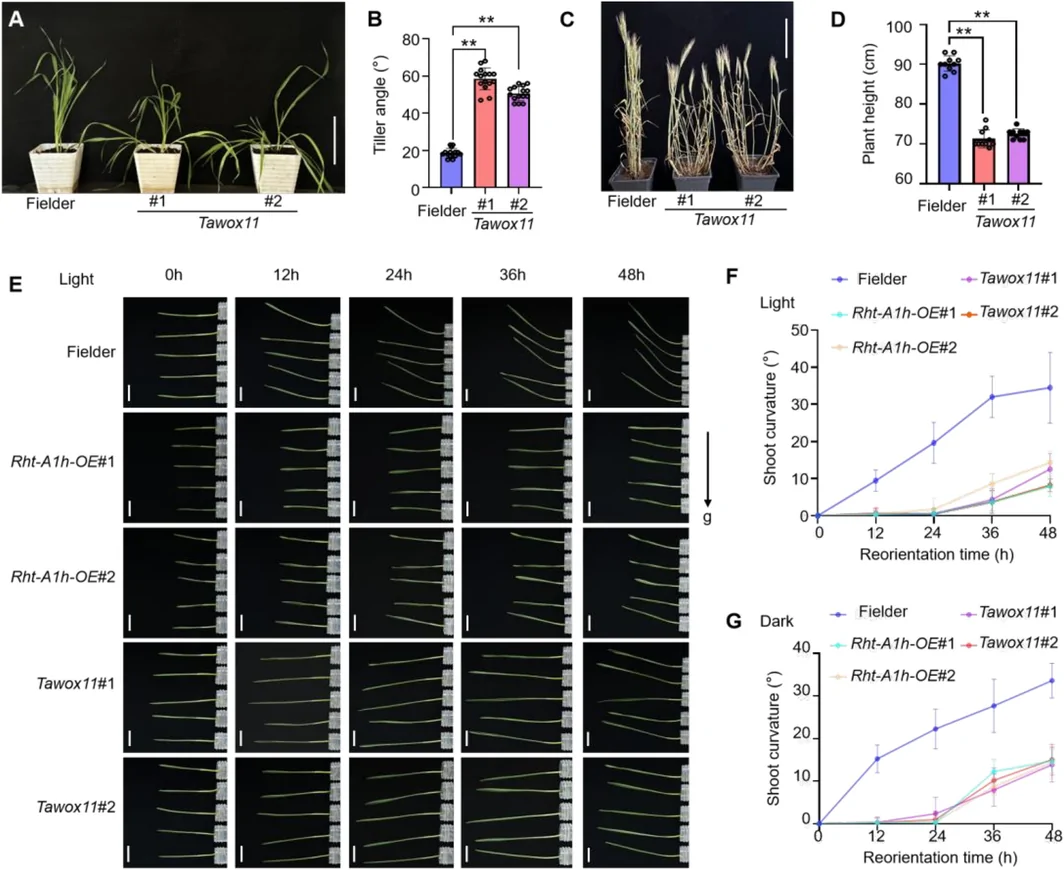
*TaWOX11* regulates tiller angle and plant height and its mutation impairs shoot gravitropism in wheat. (A) *Tawox11* (#1, #2) at the jointing stage, showing increased tiller angle and dwarfism compared to wild‐type Fielder. Scale bar = 20 cm. (B) Quantitative comparisons of tiller angle between Fielder and *Tawox11* plants in (A). Data are mean ± SD; **p < 0.01 (Student's *t*‐test). (C) *Tawox11* (#1, #2) at the grain filling stage, showing increased tiller angle and dwarfism compared to wild‐type Fielder. Scale bar = 20 cm. (D) Quantitative comparisons of plant height between Fielder and *Tawox11* plants in (C). Data are mean ± SD; **p < 0.01 (Student's *t*‐test). (E) Shoot curvature of Fielder, *Rht‐A1h‐OE* and *Tawox11* seedlings after 48 h of gravistimulation under light conditions. Scale bar = 2 cm. (F) Kinetics of shoot curvature under light conditions. (G) Kinetics of shoot curvature under dark conditions. Data in (F) and (G) represent mean ± SD; *n* = 30 individual plants.

### 2.8 Ectopic Expressing *Rht‐A1h* and *TaPROG1* Mimics *TaWOX11* Deletion to Impair Gravitropism in Wheat

Finally, we systematically analysed the shoot gravitropic responses of *Rht‐A1h*‐, *TaPROG1*‐ and *TaWOX11*‐related genetic materials to evaluate their functional relevance in tiller angle regulation. First, we compared the shoot gravitropism of *Rht‐A1h‐OE* and *Tawox11* seedlings. Following gravistimulation, *Rht‐A1h‐OE* seedlings showed significantly reduced shoot curvature compared to wild‐type under both light and dark conditions (Figure 7E–G; Figure S14). This phenotype mirrors the attenuated shoot gravitropism observed in both *prose1* (Figure 2G–J) and *Tawox11* mutants (Figure 7E), further supporting the similar effects of *Rht‐A1h* expression and *TaWOX11* knockout on shoot gravitropism in wheat. In contrast, *Rht‐A1a‐OE* T_1_ seedlings exhibited shoot gravitropic responses comparable to wild‐type Fielder (Figure S15), confirming that overexpression of the wild‐type *Rht‐A1a* protein does not impair shoot gravitropism.

Next, we investigated whether *TaPROG1* overexpression can mimic *Rht‐A1h* overexpression or *TaWOX11* mutation in controlling shoot gravitropism. We employed the MCD‐mediated transient transformation strategy to analyse the effect of *TaPROG1* at this stage. Results confirmed that Fielder wheat seedlings expressing TaPROG1‐Flag proteins indeed exhibited significantly impaired shoot gravitropic responses relative to wild‐type controls, largely phenocopying the *Tawox11* mutant (Figures S16 and S17). Moreover, we also expressed TaPROG1‐Flag in the *Tawox11* genetic background to test potential epistatic interactions between these two components. Unexpectedly, expressing TaPROG1 in the *Tawox11* background resulted in more severe shoot gravitropic defects than those observed in *Tawox11* alone (Figures S16 and S17), suggesting that in addition to the regulatory interaction between *TaPROG1* and *TaWOX11*, *TaPROG1* also modulates shoot gravitropism through other genes and signalling pathways independent of *TaWOX11*. This finding underscores the complexity of the regulatory circuitry and supports the view that multiple genes and pathways contribute to the phenotype.

Together, these findings indicate that ectopic expression of *Rht‐A1h* and *TaPROG1* or knockout of *TaWOX11*, each confers a similarly impaired shoot gravitropic phenotype in wheat. However, the regulatory relationships among these three components in controlling shoot gravitropism are likely more complex than currently understood, as they may act both synergistically and additively.

## 3 Discussion

### 3.1 Rht‐A1h Defines a Gain‐Of‐Function Mutation in DELLA Protein With a Phenotype Similar to Green Revolution Rht‐B1b

The deployment of semi‐dwarfing alleles *Rht‐B1b* and *Rht‐D1b* was pivotal to the Green Revolution, dramatically increasing wheat yield by reducing plant height and improving lodging resistance. Notably, the A subgenome‐derived homoeolog, *Rht‐A1*, was not utilized in these breeding efforts and was considered less consequential (Peng et al. 1999). Classical Green Revolution alleles encode truncated DELLA proteins that are stable and resistant to GA‐mediated degradation, leading to constitutive repression of GA signalling and GA‐insensitive dwarfism (Zhang et al. 2019). In contrast, our study identifies *Rht‐A1h* as a semi‐dominant DELLA variant. The *prose1* phenotype is caused by a single amino acid substitution (Gly to Glu) at the third residue downstream of the core DELLA motif (D‐E‐L‐L‐A), within the N‐terminal regulatory region of Rht‐A1 (Figure 3A). Thus, while *Rht‐A1h* phenocopies the dwarfism of the Green Revolution alleles, it achieves this through a distinct molecular lesion at the A locus, underscoring the evolutionary plasticity of DELLA proteins across subgenomes.

While the Green Revolution focused primarily on height and lodging, our results—together with recently published works (Chen et al. 2026; Zhang et al. 2025)—reveal that both the classical *Rht‐B1b* and *Rht‐D1b* alleles and the newly identified *Rht‐A1h* allele are also central regulators of tiller angle. Remarkably, a parallel study independently identified the same *Rht‐A1* allele in wheat during the review process of our manuscript (Chen et al. 2026). This study established that the Rht‐A1–PROG1 module represses *LAZY1‐D* transcription. Our work provides complementary evidence that *TaWOX11* serves as an additional downstream target of this module. Rht‐A1h exhibits enhanced binding to TaPROG1 compared to Rht‐A1a, correlating with increased repression of *TaWOX11* transcription. We also observed differential binding of Rht‐A1h to other regulators including TaLAZY1, TaLAZY4, TaD14 and TaHST1L. Together, these studies indicate that the Rht‐A1–TaPROG1 module may regulate tiller angle through multiple downstream targets, with our work identifying *TaWOX11* as one such component (Figure 5A; Figure S8A,B).

The G‐to‐E substitution in Rht‐A1h is located three amino acids downstream of the core DELLA motif. Recent structural evidence from *Arabidopsis* shows that the DELLA domain directly interacts with both the GRAS domain and GID1A and that disrupting this intramolecular DELLA‐GRAS interaction weakens GID1 binding (Dahal et al. 2025). This suggests that mutations in the DELLA‐adjacent region may alter GRAS domain conformation and thereby modulate interactions with partner proteins such as TaPROG1. Whether this structural mechanism is conserved in wheat Rht‐A1 requires further investigation.

Nonetheless, our findings establish Rht‐A1h as a DELLA variant. The *Rht‐A1h* allele provides a new genetic resource and a novel dominant mechanism for simultaneously modulating plant height and canopy structure, offering fresh paradigms for designing ideal plant architecture in wheat.

### 3.2 Rht‐A1 Protein Interacts With Key Components of Tiller Angle Regulation

DELLA proteins lack DNA‐binding domains and function as transcriptional co‐regulators by interacting with a diverse array of transcription factors, thereby integrating GA signals with other developmental and environmental pathways (Feng et al. 2008; Lantzouni et al. 2020; Li et al. 2023). For instance, DELLAs interact with PIFs to coordinate light and GA responses (Li et al. 2016), with BZR1 to mediate brassinosteroid‐GA crosstalk (Li et al. 2012) and with GRF4 to balance carbon‐nitrogen metabolism (Li et al. 2018). Our protein–protein interaction screening extends this central ‘hub’ function of DELLAs to the regulation of plant architecture. We found that Rht‐A1 physically associates with multiple known regulators of tiller angle, including TaLAZY1, TaLAZY4, TaHST1L, TaPROG1 and TaD14 (Figure 5A; Figure S8A,B). Importantly, Rht‐A1a and Rht‐A1h exhibited differential interaction strengths with each of these tiller‐angle‐associated proteins. Among these, the difference in interaction strength between Rht‐A1a and Rht‐A1h with TaPROG1—a key C2H2 zinc‐finger transcription factor determining plant architecture in rice—was the most pronounced. Rht‐A1h exhibited stronger interactions with TaPROG1 compared to Rht‐A1a (Figure 5A; Figure S8A,B). This enhanced affinity provides a direct molecular explanation for the semi‐dominant nature of the *Rht‐A1h* allele and suggests that *Rht‐A1* regulates tiller angle and overall plant form by modulating the activity of its conjugators including TaPROG1.

Notably, the differential interaction pattern between Rht‐A1a and Rht‐A1h was not uniform across all tested tiller angle regulators: while most interactors showed enhanced association with the mutant protein, TaHST1L exhibited preferential interaction with the wild‐type Rht‐A1a (Figure S8B). This bidirectional shift in interaction affinities suggests that the Gly‐to‐Glu substitution in Rht‐A1h does not simply increase or decrease binding universally, but rather remodels the protein's interaction landscape in a partner‐specific manner. Such specificity implies that the mutation may alter the conformation or surface charge distribution of the DELLA protein in ways that differentially affect binding interfaces with distinct interactors. This nuanced remodelling of protein interaction stands in contrast to the classical Rht‐B1b/D1b mutations, which merely abolish protein degradation and lead to protein accumulation for a strengthened but not‐selective genetic effect. The Rht‐A1h mechanism thus represents a more precise genetic perturbation—one that fine‐tunes rather than uniformly enhances protein function—potentially enabling more precise control over downstream developmental outputs.

The Rht‐A1h mutation appears to achieve its effects by precisely altering a specific protein interaction interface. This mechanism could potentially enable the refinement of plant architecture—reducing height and adjusting tiller angle—without the associated yield penalties, representing a more sophisticated approach to trait engineering. It should be noted that *Rht‐A1h*‐OE lines exhibited a stronger phenotype than the *prose1* mutant, which is expected due to the constitutive *Ubi* promoter driving higher expression than the endogenous gene copy. This supports the gain‐of‐function nature of *Rht‐A1h*. However, one point should also be noted: the Rht‐A1h mutation has not only altered its interaction with TaPROG1, which was extensively analysed in this study, but also modified its association with multiple other tiller angle regulators (Figure 5A; Figure S8A,B). This raises the important consideration that the Rht‐A1h‐triggered prostrate phenotype is unlikely to be solely attributable to TaPROG1, but likely reflects the combined contributions of multiple differentially interacting partners. Therefore, we assume a model in which Rht‐A1h acts as a signalling hub, with its mutation altering the balance of interactions across a set of physical interacting partners including TaPROG1, TaLAZY1, TaLAZY4, TaD14 and others, collectively contributing to the observed prostrate growth habit. Future efforts should focus on systematically dissecting the genetic effects of these individual interactions and their relative contributions to tiller angle regulation. Distinguishing the functional differences among these interactors will be essential for more precisely controlling the phenotypic outcomes of Rht‐A1h‐based breeding strategies and for understanding how DELLA proteins coordinate complex architectural traits through their multifaceted interaction.

### 3.3 *TaWOX11* Is a Critical Downstream Target in Wheat Gravitropism and Plant Architecture

Meristems are fundamental to plant architecture, responsible for generating the diversity of plant forms (Dresselhaus et al. 2025). Among key meristem regulators, *WUS* and its homologues in the *WOX* family encode homeodomain transcription factors that maintain the stem cell niche in both the shoot apical meristem (SAM) and root apical meristem (RAM). These genes are crucial for post‐embryonic development, organogenesis and the shaping of lateral organs (Jha et al. 2020; Wu et al. 2024), acting as central mediators of auxin signalling during gravitropic responses (Cheng et al. 2018; Hu et al. 2020; Wang et al. 2014). Our study reveals *TaWOX11* as one of the critical downstream effectors in the Rht‐A1h–TaPROG1 regulatory pathway. Our data show that Rht‐B1b, like Rht‐A1h, represses *TaWOX11* expression and acts synergistically with TaPROG1 to enhance this repression (Figure S8C). This suggests that the regulatory effect on *TaWOX11* is not unique to the A‐subgenome DELLA allele but may be shared with at least one other DELLA variant. Whether this conservation extends to other DELLA alleles or subgenomes will require further investigation. Nonetheless, these results raise the possibility that the enlarged tiller angle observed in *Rht‐B1b*‐OE lines could be partially attributed to *TaWOX11* repression, in addition to the known effects of *Rht‐B1b* on plant height.

In rice, *WOX11* and its homologue *WOX6* have been shown to function redundantly in regulating tiller angle and crown root development through auxin‐mediated pathways (Zhang et al. 2018). However, our results reveal a different and potentially more direct role for *TaWOX11* in wheat. The strong repression of *TaWOX11* in *Rht‐A1h‐OE* plants (Figure 6C,D), together with the in vivo evidence showing reduced *TaWOX11* expression in *TaPROG1* overexpression lines (Figure 6G), as well as direct TaPROG1 binding to the *TaWOX11* promoter (Figure 6E), establishes a direct molecular link between the Rht‐A1h–TaPROG1 complex and *TaWOX11* regulation.

Phenotypic characterization of CRISPR/Cas9‐generated *Tawox11* null mutants in wheat provides compelling evidence for its essential function. These mutants phenocopied the increased tiller angle and impaired shoot gravitropism of *Rht‐A1h‐OE* lines (Figure 7), confirming *TaWOX11* as a major positive regulator of shoot gravitropism in wheat. Consistent with this, *TaPROG1* overexpression lines exhibited enlarged tiller angles (Figure 5D–F) and seedlings transiently expressing TaPROG1‐Flag showed partially impaired shoot gravitropic responses (Figures S16 and S17), further supporting the functional relationship between *TaPROG1* and *TaWOX11* in this regulatory pathway. This indicates that in hexaploid wheat, *TaWOX11* acts as a major and positive regulator of shoot gravitropism, and its suppression is a key event in the pathway through which the Rht‐A1h‐TaPROG1 complex promotes prostrate growth. This functional shift from the redundant mechanism observed in rice emphasizes the evolutionary rewiring of developmental signalling in wheat and establishes *TaWOX11* as an essential integration node under the cooperative control of Rht‐A1 and TaPROG1.

Although we were unable to obtain a *Tawox11‐A* single mutant due to the high mutagenesis efficiency of CRISPR/Cas9 at all three homoeologous loci simultaneously, our observation that all three *TaWOX11* homoeologs are downregulated in both *Rht‐A1h‐OE* and *TaPROG1‐OE* lines supports a model in which the three homoeologs contribute redundantly to tiller angle regulation. The phenotypic difference between wheat *Tawox11* triple mutants and rice *wox11* single mutants is noteworthy. In rice, *WOX6* and *WOX11* function redundantly in regulating tiller angle, with single mutants of either gene showing no obvious phenotype. By contrast, our wheat *Tawox11* triple mutants—without mutation in *TaWOX6*—already exhibited pronounced enlarged tiller angles. This apparent divergence from the rice model may reflect species‐specific differences in the roles of *WOX6* and *WOX11* in tiller angle regulation between the two species. Complementation of the *Tawox11* triple mutant with *TaWOX11‐A* remains an important piece of evidence for supporting the in vivo genetic effect of *TaWOX11*; however, this experiment was not completed in the current study due to the extremely time‐consuming nature of combining *TaWOX11‐A* expression with the *Tawox11* triple mutant background, and we have noted this as a priority for future work.

Whether the reduced plant height in *Tawox11* mutants is a direct consequence of *TaWOX11* loss or an indirect consequence of altered tiller angle also remains unclear. While we cannot formally exclude an indirect relationship, the pleiotropic effects observed in the *Tawox11* triple mutants indicate that *TaWOX11* might regulate these two traits through at least partially independent mechanisms, a possibility that warrants further investigation.

We must also remain cautious and rigorous in defining the precise contribution of *TaWOX11* to the Rht‐A1h–TaPROG1 module‐regulated signalling pathways. The observation that expressing *TaPROG1* in the *Tawox11* genetic background resulted in more severe shoot gravitropic defects than those observed in *Tawox11* alone (Figures S16 and S17) clearly indicates that *TaPROG1* modulates shoot gravitropism not solely through *TaWOX11*, but also via additional, *TaWOX11*‐independent pathways. In support of this, a parallel study recently published reported that the same Rht‐A1 mutant form (identical to Rht‐A1h identified in this study), in association with TaPROG1, represses another downstream gene, *TaLAZY1* (*TaLA1*), a known regulator of tiller angle (Chen et al. 2026). Their work, together with ours, suggests that the Rht‐A1h–TaPROG1 module simultaneously regulates multiple downstream genes, which collectively contribute to the Rht‐A1h mutation‐triggered prostrate phenotype. This reveals an important layer of regulatory complexity: while *TaWOX11* serves as a critical downstream effector, it is not the sole contributor underlying the entire genetic effect of Rht‐A1h–TaPROG1 module on shoot architecture. Rather, *TaWOX11* should be considered as one of the representative downstream targets that would be employed to illustrate the differential regulatory effects between Rht‐A1a–TaPROG1 and Rht‐A1h–TaPROG1, with other contributors such as *TaLA1* also playing important roles in the overall phenotypic outcome.

This genetic evidence points to a more plausible scenario: The Rht‐A1h–TaPROG1 module targets multiple genes converging on shoot gravitropic responses, and *TaWOX11* functions within broader transcriptional pathways rather than as a linear terminal output. The partial epistasis observed between *TaPROG1* and *TaWOX11* suggests a branched regulatory architecture in which TaPROG1 operates through both *TaWOX11*‐dependent and *TaWOX11*‐independent branches that collectively shape the shoot gravitropic response. Due to the long duration required for stable wheat transformation and progeny selection, we were unable to perform the complementation and epistasis tests within the current study; these remain important directions for future investigation.

### 3.4 Potential Application of the *Rht‐A1h* Mutation for Breeding Wheat Varieties With Improved Canopy Structure

Traditional wheat cultivars often exhibit overly erect phenotypes, resulting in dense canopies with poor light penetration into the middle and lower layers, especially under high‐density planting conditions. This suboptimal architecture severely limits the yield potential of modern intensive farming systems by reducing photosynthetic efficiency and promoting disease development in the lower canopy. Conversely, an excessively prostrate growth habit can also compromise light interception efficiency. Therefore, optimizing canopy structure through precise modulation of tiller angle and plant height represents a crucial strategy for breaking the current yield plateau by maximizing light energy utilization efficiency.

The *Rht‐A1h* allele presents a unique opportunity to achieve this ‘ideal plant type’ canopy architecture. It confers a moderate semi‐dwarf stature combined with a looser, more open architecture at the heading stage (Figure 1B), which could significantly improve light distribution within the canopy and enhance photosynthetic efficiency throughout the canopy profile. Notably, the planting density‐sensitive nature of the *prose1* phenotype (Figure 1C; Figure S1) suggests that *Rht‐A1h*‐mediated architectural modulation is particularly responsive to environmental cues, offering built‐in flexibility for different planting systems. This density‐dependent plasticity represents a significant advantage over conventional dwarfing genes, allowing for optimal performance across varying agronomic practices.

The fact that the A subgenome‐derived *Rht‐1* locus was largely overlooked during the Green Revolution means it represents an untapped reservoir of genetic diversity. Unexploited allelic variants like *Rht‐A1h* could be a treasure trove for future breeding breakthroughs. By leveraging such alleles, breeders can precisely sculpt plant architecture, moving beyond the limitations of classical dwarfing genes to develop new cultivars with optimized canopies primed for higher yields.

### 3.5 Concluding Perspectives

This study identifies a semi‐dominant allele and delineates a regulatory pathway from protein interaction to gene expression regulation and phenotypic manifestation. The Rht‐A1h‐TaPROG1‐*TaWOX11* module represents a sophisticated mechanism for coordinating plant height and architecture in response to developmental and environmental cues (Figure S18). Future research should explore several important aspects: First, how this module interacts with other known regulatory pathways, particularly those involving phytohormones such as auxin and strigolactones that are known to affect tiller angle. Second, detailed investigations into how this system responds to different environmental conditions, especially planting density and light quality, would provide valuable insights for developing climate‐resilient varieties. Third, exploration of potential epistatic interactions between *Rht‐A1h* and other dwarfing genes could reveal opportunities for synergistic architectural optimization.

## 4 Materials and Methods

### 4.1 Generation of an EMS‐Mutagenized Population

For ethyl methanesulfonate (EMS) mutagenesis, approximately 1 kg of pure‐line Liangxing 99 (LX99; genotype: *Rht‐A1a/Rht‐B1a/Rht‐D1b*) seeds were surface‐sterilized with 0.1% HgCl_2_ for 15 s, followed by three rinses with sterile ultrapure water. The sterilized seeds were imbibed in sterile ultrapure water at 20°C for 8 h, with rinsing every 2 h. After imbibition, seeds were spread on moistened filter paper until radicle emergence. The germinating seeds were then rinsed three times with sterile ultrapure water and air‐dried in a cool place.

The air‐dried seeds were immersed in 0.6% EMS prepared in phosphate buffer and incubated at 20°C with gentle shaking (30 rpm) for 6 h. The mutagenesis was terminated by adding 5% sodium thiosulfate (Na_2_S_2_O_3_) solution. Finally, the treated seeds were rinsed three times with sterile ultrapure water and air‐dried in a cool place prior to sowing.

The EMS‐treated LX99 seeds were sown at the China Agricultural University‐Jize Experimental Station (Jize County, Handan City, Hebei Province, China). Seeds were planted in 1.5 m‐long rows with 20 cm spacing between rows. At maturity, approximately 1200 individual plants (M_1_ generation) were harvested. The harvested M_1_ plants were subsequently advanced through single‐seed descent until the M_4_ generation and beyond to ensure genetic stability prior to phenotypic screening. Beginning at the mid‐grain‐filling stage, we evaluated and documented a comprehensive suite of agronomic traits for each putative mutant, including growth period, plant height, tillering capacity, plant architecture, flag leaf morphology (leaf length and width) and spike characteristics (spike length and spikelet number).

### 4.2 Plant Materials and Growth Conditions

The *prose1* mutant was selected from the EMS‐mutagenized population. F_1_ plants were produced by crossing the *prose1* mutant with either LX99 or the wheat cultivar Jing411 (J411; genotype: *Rht‐A1a/Rht‐B1a/Rht‐D1a*). F_2_ segregating populations were obtained by self‐crossing F_1_ plants. An F_2:3_ population was subsequently developed by self‐pollination of F_2_ plants heterozygous within the candidate genomic region and used for fine‐mapping of the causal mutation. All plants for phenotypic evaluation were grown under field conditions at the Shangzhuang Experimental Station (China Agricultural University, Beijing, China; 40° N, 116° E). Seeds were sown in 1.5 m‐long rows with 20 cm spacing between rows.

For genetic transformation and gene editing experiments, the spring wheat cultivar Fielder (genotype: *Rht‐A1a/Rht‐B1b/Rht‐D1a*) was used. Transgenic plants were grown in a greenhouse at China Agricultural University under the following controlled conditions: 60% relative humidity, day/night temperatures of 25°C/20°C and a light intensity of 3000 lx (provided by Master GreenPower, Philips). *Nicotiana benthamiana* plants and hydroponic assays were maintained in a greenhouse under a 16‐h light/8‐h dark photoperiod at 22°C.

### 4.3 MCD‐Mediated Seed Transient Transformation

Transient transformation of wheat seeds was performed using the modified carbon dot (MCD)‐mediated method as described previously (She et al. 2025). Approximately 20–30 selected seeds were completely submerged in 2–3 mL of MCD solution per tube, containing 20 μg/μL MCDs, 10 ng/μL plasmid DNA, 0.5% Tween‐20 and 10 mM MES buffer (2‐(N‐morpholino) ethanesulfonic acid, pH 5.8). The tubes were incubated at 25°C for 3 h and then placed at 4°C overnight.

The following day, the tubes were removed and allowed to reach room temperature on the bench, followed by incubation in a 37°C water bath for an additional 30 min. Subsequently, two rounds of vacuum treatment were applied for 5 min each. After vacuum treatment, the tubes were incubated at 25°C for 3 h and then returned to 4°C for overnight incubation. On the third day, the MCD‐treated seeds were thoroughly washed 3–4 times with autoclaved distilled water, then placed in petri dishes lined with two layers of moistened qualitative filter paper for germination at room temperature. This MCD‐mediated system enables efficient delivery of DNA plasmids into wheat seeds and generates whole seedlings containing the transferred DNA constructs.

### 4.4 Phenotypic Analysis

Agronomic traits of various plant materials were evaluated under field conditions at the Shangzhuang Experimental Station, Beijing, China (40° N, 116° E). Tiller angle was measured at the jointing stage (approximately 20–30 cm in plant height) and also at the grain filling stage for phenotypic comparisons. For each plant, the horizontal distance between the outermost tiller and the central vertical axis was recorded, and the tiller angle was calculated using the Pythagorean theorem. Based on the phenotypic distribution, individual plants were classified into three categories: erect type (<20°, resembling the parental lines J411 and LX99), intermediate type (20°–45°) and prostrate type (>45°, resembling the *prose1* mutant).

Prior to harvest, the following traits were recorded for each individual plant: plant height (PHT), spike length (SL), effective tiller number per plant, spikelet number per spike and the lengths of the first to fifth internodes (counted from the top to the bottom) of the main tillers. Grains from the main tillers were collected at maturity and bulk‐harvested for the measurement of thousand‐grain weight (TGW), grain length (GL) and grain width (GW). TGW, GL and GW were assessed using a camera‐assisted phenotyping system (Wanshen Detection Technology, Hangzhou, China). All measurements were conducted with three biological replicates.

### 4.5 Cell Size Analysis

At the heading stage, the middle segments of the first and second internodes (from the top downward) were collected from both the wild‐type LX99 and the *prose1* mutant for histological examination. The samples were processed as follows: (1) Fixation: Samples were fixed in FAA solution (10% formaldehyde, 50% alcohol, 5% acetic acid and 35% water, v/v) overnight at 4°C. (2) Embedding: Fixed tissues were embedded in paraffin. (3) Dehydration and decolorization: The embedded samples were dehydrated and decolorized according to established methods (Chai et al. 2022). (4) Sectioning: Transverse sections of 4 μm thickness were prepared using a Leica Ultracut rotary microtome (Leica Biosystems). (5) Staining: Sections were mounted on glass slides and stained with 1% safranin O and 0.5% fast green (G1031, Servicebio). (6) Imaging: Images of stained sections were acquired using a Nikon Eclipse E100 microscope equipped with a DS‐U3 camera system. (7) Measurement: The cell lengths were measured using CaseViewer 2.3 software (3DHISTECH).

### 4.6 BSR‐Seq Analysis

Leaf tissues at the seedling stage were collected from 30 homozygous *prose1* mutants and 30 homozygous wild‐type plants to construct two bulk samples for RNA extraction. Total RNA was isolated using TRIzol reagent (Invitrogen) following the manufacturer's instructions. RNA‐seq libraries were prepared and sequenced on the Illumina NovaSeq 6000 platform in paired‐end mode. Raw reads were quality‐trimmed using Trimmomatic v0.32 with default parameters (Bolger et al. 2014). High‐quality reads were then aligned to the IWGSC RefSeq v1.0 genome of Chinese Spring using STAR v2.4.0j (Dobin et al. 2013). Variant calling was performed with the HaplotypeCaller module in GATK v3.2–2 (McKenna et al. 2010). To identify candidate regions linked to the mutant phenotype, variants were filtered based on an allele frequency difference (AFD) > 0.8 and a Fisher's exact test *p*‐value < 1 × 10^−10^.

### 4.7 Genome Resequencing

Genomic DNA was extracted from leaves of wild‐type LX99 and the *prose1* mutant using a modified CTAB method (Murray and Thompson 1980). Sequencing was performed on the Illumina NovaSeq 6000 platform, generating 2 × 150 bp paired‐end reads with approximately 15× coverage of the assembled genome. High‐quality reads were aligned to the IWGSC RefSeq v1.0 reference genome of Chinese Spring using Burrows‐Wheeler Aligner (BWA v0.7.15) with default parameters (Li and Durbin 2009). Variant calling, including SNP and insertion/deletion (InDel) identification, was conducted using the HaplotypeCaller module in the GATK toolkit.

### 4.8 Map‐Based Cloning

An F_1_ population was generated by crossing the wheat cultivar LX99 with the *prose1* mutant, and a subsequent F_2_ population consisting of 242 individuals was developed through self‐pollination for genetic analysis and initial mapping of the candidate gene. Bulked segregant RNA‐seq (BSR‐seq) analysis was performed to identify candidate SNPs associated with tiller angle, enabling chromosomal localization of the target gene. For high‐resolution fine‐mapping, an additional F_1_ population was generated by crossing another wheat cultivar, Jing411 (J411), with the *prose1* mutant. Self‐pollination of these F_1_ plants produced an F_2_ population of 436 individuals, from which heterozygous recombinant plants were selected and selfed to generate a large F_2:3_ population comprising 1048 individual lines. Phenotypic evaluation of both the F_2_ and F_2:3_ populations derived from the J411 × *prose1* cross was conducted to ensure mapping accuracy. Genome resequencing data from the *prose1* mutant, LX99 and J411 were used to detect SNP variations. Molecular markers were developed based on these polymorphisms using the Chinese Spring reference genome (IWGSC RefSeq v2.0). Linkage analysis was conducted with JoinMap 4.0 (http://www.kyazma.nl/index.php/mc.JoinMap/sc.General).

### 4.9 Gene Cloning and Sequence Analysis

The coding sequence (CDS) of *Rht‐A1a* was cloned from the cDNA of LX99, while the CDS of *Rht‐A1h* was obtained from the cDNA of *prose1*. Specific primer pairs (Data S5) were designed to amplify the promoter and full‐length coding sequences of candidate genes. PCR was performed using Tks Gflex DNA polymerase (TaKaRa) under the following conditions: 94°C for 5 min; 35 cycles of 98°C for 10 s, 58°C for 15 s and 68°C for 1 min; followed by a final extension at 68°C for 5 min. Amplified products were sequenced. Nucleotide and amino acid sequences were analysed using DNAMAN and the Conserved Domain Search online tool (NCBI). The analysed sequence included the open reading frame (ORF) and 2‐kb upstream of the translation start codon.

### 4.10 RNA‐Seq and Data Analysis

Tiller bases from wild‐type Fielder and *Rht‐A1h*‐overexpressing (*Rht‐A1h‐OE*) transgenic plants at the jointing stage were collected for RNA sequencing. Three biological replication samples were collected. Sequencing libraries were prepared using TruSeq Poly‐A library reagents and sequenced on an Illumina HiSeq 2500 platform. After quality trimming, clean reads were aligned to the IWGSC RefSeq v1.1 wheat genome. Differentially expressed genes (DEGs) were identified using the thresholds |log_2_(fold change)| > 1 and adjusted *p*‐value < 0.05. Gene Ontology (GO) enrichment analysis was performed using Triticeae‐GeneTribe (http://wheat.cau.edu.cn/TGT/).

### 4.11 RNA Extraction and qRT–PCR

Total RNA was extracted with TRIzol reagent (Invitrogen), and cDNA was synthesized using HiScript II Q RT SuperMix (Vazyme). Quantitative real‐time PCR (qRT‐PCR) was performed with qPCR SYBR Green Master Mix (Vazyme), using *Actin* as the internal control. The PCR conditions were: 95°C for 4 min; 40 cycles of 95°C for 10 s, 58°C for 20 s and 72°C for 20 s. Reactions were run on a Bio‐Rad C1000 system. Transcript levels were normalized to *Actin* and calculated using the $2^{-\Delta \Delta C_{t}}$ method (Livak and Schmittgen 2001). All primers are listed in Data S5.

### 4.12 Genetic Transformation in Bread Wheat

For overexpression, the coding sequence (CDS) of *Rht‐A1h* was amplified using Phanta Max Super‐Fidelity DNA Polymerase (P515, Vazyme Biotech) and cloned into the *BamHI* site of the *pWMB110* vector. Promoters of *Rht‐A1* and *Rht‐B1* were similarly amplified and inserted into *pWMB110* with the *RUBY* reporter using *HindIII* and *AgeI*. For CRISPR/Cas9 knockout, sgRNAs targeting *TaWOX11‐A/B/D* were designed using E‐CRISPR (http://www.e‐crisp.org/E‐CRISP/) and cloned into the *pBUE411* vector. Constructs were transformed into *Agrobacterium tumefaciens* EHA105 and introduced into Fielder. Transgenic plants were screened via PCR and expression analysis. For CRISPR/Cas9‐mediated mutagenesis of *TaWOX11*, a single vector carrying two sgRNAs targeting conserved regions of *TaWOX11‐A*, *‐B* and *‐D* was constructed and introduced into wheat (Fielder) via Agrobacterium‐mediated transformation. A total of 8 independent transgenic lines were obtained. Genotyping by sequencing confirmed that sgRNA1 induced mutations at all three homoeologous loci, whereas sgRNA2 showed no detectable editing. Cas9‐free T_3_ progeny were selected by PCR using Cas9‐specific primers and used for phenotypic analysis.

### 4.13 Analysis of Shoot Gravitropism

Shoot gravitropic responses were assayed as described (Li et al. 2007) with modifications. Seeds were germinated on 0.4% agar in tubes at 22°C for 4 days, then reoriented by 90°. Curvature was measured at indicated times.

### 4.14 Electrophoretic Mobility Shift Assay

Oligonucleotides were labelled with biotin at the 5′‐end (Invitrogen). EMSA was performed using the LightShift Chemiluminescent EMSA Kit (Thermo Fisher Scientific, #20148). Each 20 μL reaction contained 20 ng purified protein, 5 ng annealed biotin‐labelled probe, 2 μL binding buffer, 1 μL 50% (v/v) glycerol, 1 μL poly(dI‐dC) (1 μg/μL), 1 μL 100 mM MgCl_2_, 1 μL 1 M KCl, 0.2 μL 200 mM EDTA and 1 μL 1% NP‐40. After incubation at 25°C for 20 min, samples were resolved on a native polyacrylamide gel. Biotin‐labelled DNA was detected following the manufacturer's protocol, and images were acquired using a ChemiScope 6100 system (Qinxiang, China). All oligonucleotide probes and primers are listed in Data S5.

### 4.15 Split‐Luciferase Complementation Imaging Assay

The CDSs of the genes of interest were individually cloned into the *KpnI*/*SalI* sites of the *pCAMBIA1300‐nLUC* vector and the *KpnI*/*BamHI* sites of the *pCAMBIA1300‐cLUC* vector (Chen et al. 2007). The resulting constructs were introduced into *Agrobacterium tumefaciens* strain GV3101 and co‐infiltrated into leaves of *Nicotiana benthamiana*. At approximately 36 h post‐infiltration, luciferase (LUC) signals in the infiltrated areas were visualized and quantified using a NightSHADE LB 985 plant imaging system (Berthold Technologies).

### 4.16 In Vitro Pull‐Down Assay

The coding sequences of *Rht‐A1a* and *Rht‐A1h* were amplified and cloned in‐frame into the *EcoRI*/*XbaI* sites of the *pMAL‐c2x* vector (NEB, #E8000S) to generate MBP fusion proteins. The ORF of *TaPROG1* was cloned into the *EcoRI*/*SalI* sites of the *pET‐32a* vector (Novagen) to produce a His‐tagged fusion protein. Recombinant proteins were expressed in *E. coli* Rosetta (DE3) cells induced with 0.5 mM IPTG in LB medium at 16°C overnight and purified according to the manufacturers' protocols.

For the pull‐down assay, bacterial lysates containing the expressed proteins were incubated with high‐affinity amylose resin (NEB, #E8021L) in Tris buffer (25 mM Tris–HCl, pH 7.5, 50 mM NaCl, 1 mM DTT) for 5 h at 4°C. The resin was washed eight times with 1 mL of Tris buffer supplemented with 1% (v/v) Triton X‐100. Bound proteins were eluted in 100 μL of 2× SDS loading buffer (100 mM Tris–HCl pH 6.8, 20% glycerol, 4% SDS, 10% β‐mercaptoethanol) by boiling at 100°C for 10 min. Eluates were separated by 10% SDS‐PAGE, and immunoblotting was performed using anti‐MBP (1:5000, TransGen Biotech, #HT701‐01) or anti‐His (1:5000, TransGen Biotech, #HT501‐01) antibodies. Three biological replicates were conducted. All primers used are listed in Data S5.

### 4.17 Bimolecular Fluorescence Complementation Assay

The full‐length ORF of *Rht‐A1a* was cloned into the *BamHI*/*XbaI* sites of the N‐terminal YFP (nYFP) vector, and the full‐length ORF of *TaPROG1* was cloned into the *BamHI*/*XbaI* sites of the C‐terminal YFP (cYFP) vector. The resulting constructs were introduced into *Agrobacterium tumefaciens* strain GV3101. Bacterial cultures carrying the nYFP and cYFP fusion constructs were co‐infiltrated into leaves of *Nicotiana benthamiana*. Yellow fluorescent protein (YFP) signals were observed 48 h after infiltration using a confocal laser scanning microscope (LSM 880; Carl Zeiss) with the following settings: excitation at 488 nm, laser intensity set to 3.98%, emission collection bandwidth of 484–543 nm, and photomultiplier gains between 600 and 800 V. The experiment included three independent biological replicates. All primers used are listed in Data S5.

### 4.18 Dual‐Luciferase Transcriptional Activity Assay

To generate the reporter construct, a 2‐kb fragment upstream of the start codon (ATG) of the A subgenome‐derived *TaWOX11* (*TaWOX11‐A*) was amplified and inserted into the *HindIII*/*KpnI* site of the *pGreenII 0800‐LUC* vector to generate the reporter construct *TaWOX11‐A*_*pro*_ *::LUC* (Hellens et al. 2005). The CDSs of *Rht‐A1a*, *Rht‐A1h* and *TaPROG1* were cloned into the *XbaI* site of the *pCAMBIA1300‐GFP* vector to generate the effector constructs *Rht‐A1a‐GFP*, *Rht‐A1h‐GFP* and *TaPROG1‐GFP*. The reporter and effector plasmids were introduced into *Agrobacterium tumefaciens* strain GV3101 and co‐infiltrated into leaves of *Nicotiana benthamiana*. Leaf samples were collected approximately 36 h after infiltration. Firefly luciferase (LUC) activity and *Renilla* luciferase (REN) activity were measured using the Dual‐Luciferase Reporter Assay System (Promega) according to the manufacturer's protocol. Normalized luciferase activity was calculated as the ratio of LUC to REN luminescence. Data are presented as mean values from three independent biological replicates. All primer sequences used in this assay are listed in Data S5.

### 4.19 Accession Numbers

All genes' information used in this article can be found in the GeneBank/EMBL data libraries (http://plants.ensembl.org/index.html) under the following accession numbers: *Rht‐1* (*TraesCS4A02G271000*, *TraesCS4B02G043100* , *TraesCS4D02G040400*), *TaPROG1* (*TraesCS5A02G511400*), *TaLAZY1* (*TraesCS6D02G396500*), *TaLAZY4* (*TraesCS1B02G266300*), *TaHST1L* (*TraesCS5A02G316400*), *TaD14* (*TraesCS4B02G258200*), *TaWOX6* (*TraesCS4A02G130200*, *TraesCS4B02G174400*, *TraesCS4D02G176400*), *TaWOX11* (*TraesCS2A02G100700*, *TraesCS2B02G117900*, *TraesCS2D02G100200*), *Rht‐A1h* (BankIt3068611 Seq PZ205830). Files of raw sequence reads generated in this study were deposited in the BIG Data Center (https://ngdc.cncb.ac.cn/gsub/) under BioProject ID PRJCA045654.

## Author Contributions

Jie L., Q.S. and Z.N. conceived and designed the study. Jie L. supervised the research. C.D. performed the majority of the experiments. D.D. generated the *Tawox11* mutant plants. Y.L., S.J. and Z.Z. carried out genotypic and phenotypic characterization and initial gene mapping. Y.W. analysed the transcriptome data. C.D., D.D., Z.Z., S.J., S.Z., X.W., M.X., Y.Y. and L.C. contributed to data analysis and interpretation. Jie L. and C.D. wrote the manuscript. Q.S. and Z.N. supervised the project and edited the manuscript. All authors reviewed and approved the final version.

## Funding

This work was supported by the National Natural Science Foundation of China, 32372120; the Biological Breeding‐National Science and Technology Major Project, 2023ZD0402402; National Key Research and Development Program of China, 2022YFF1003401.

## Conflicts of Interest

The authors declare no conflicts of interest.

## Supporting information


**Figure S1:** Phenotypic characterization of the *prose1* mutant (prostrate and semi‐dwarf) under different planting densities.
**Figure S2:** Map‐based cloning of the gene associated with the *prose1* mutation.
**Figure S3:** Sequence alignment of *Rht‐A1a* and *Rht‐A1h*.
**Figure S4:** Maximum tiller inclination angles of *Rht‐A1h*‐OE and wild‐type Fielder at the jointing stage.
**Figure S5:** Phenotypic analysis of *Rht‐A1a*‐OE T_1_ transgenic lines and *Rht‐B1* expression in *Rht‐B1a*‐OE and *Rht‐B1b*‐OE lines.
**Figure S6:** Overexpression of *Rht‐B1b*, but not *Rht‐B1a*, impairs shoot gravitropism in wheat.
**Figure S7:** qRT‐PCR analysis of *Rht‐A1*, *Rht‐B1* and *Rht‐D1* expression and *RUBY* reporter assay showing *Rht‐A1*and *Rht‐B1* spatial expression patterns.
**Figure S8:** Comparison of interaction strengths between Rht‐A1a/Rht‐A1h and tiller angle regulators, and LCI analysis of Rht‐B1a/B1b interaction with TaPROG1.
**Figure S9:** AlphaFold‐based structural predictions of Rht‐A1h and Rht‐A1a.
**Figure S10:** Phenotypic analysis of *Rht‐A1a*‐OE, *Rht‐A1h*‐OE and *TaPROG1*‐OE transgenic wheat lines.
**Figure S11:** Prediction and validation of TaPROG1 binding to *TaWOX11* promoters.
**Figure S12:** CRISPR/Cas9‐mediated mutagenesis of *TaWOX11* homoeologs in wheat.
**Figure S13:** Phenotypic analysis of *TaWOX11* mutants.
**Figure S14:** Both *Rht‐A1h* overexpression and *TaWOX11* mutation partially attenuate shoot gravity‐sensing response in wheat.
**Figure S15:** Shoot gravitropism analysis of *Rht‐A1a*‐OE T_1_ seedlings compared to wild‐type Fielder.
**Figure S16:** *TaPROG1* negatively regulates shoot gravitropism partially through *TaWOX11* under light conditions.
**Figure S17:** *TaPROG1* negatively regulates shoot gravitropism partially through *TaWOX11* under dark conditions.
**Figure S18:** A working model contrasting wild‐type *Rht‐A1a* and mutant *Rht‐A1h* in regulating wheat plant height and tiller angle.

**Data S1:** Segregation analysis of tiller angle in the F_2_ population derived from the cross between Jing411 (J411) and *prose1* or between Liangxing99 (LX99) and *prose1*.
**Data S2:** Differentially expressed genes (DEGs) in *Rht‐A1h‐OE* relative to that in wild‐type Fielder.
**Data S3:** Gene ontology (GO) enrichment analysis of the upregulated DEGs in *Rht‐A1h‐OE* versus Fielder.
**Data S4:** GO enrichment analysis of the downregulated DEGs in *Rht‐A1h‐OE* versus Fielder.
**Data S5:** Primer sequences used for map‐based cloning, gene expression analysis and vector construction.

## Data Availability

All data are presented as figures and tables. The data that support the findings of this study are available in Figures S1–S18 and Data S1–S5 of this article.
