# Supplementary material for: A Gain‐of‐Function Mutation in DELLA Selectively Alters Protein Interactions to Regulate Wheat Tiller Angle and Plant Height
Source: Plant Biotechnol J. 2026 Oct 8:10.1111/pbi.70763. Online ahead of print. doi: 10.1111/pbi.70763 (PMC13650195; doi:10.1111/pbi.70763)
Supplement: Supplementary file 1 [file PBI-9999-0-s001.docx]

**Supplementary Information**

**Supplementary Figure S1. Phenotypic characterization of the *prose1* mutant (prostrate and semi-dwarf) under different planting densities.**

Plants were photographed at the jointing stage. Planting densities: high (0.1 m row spacing), medium (0.2 m row spacing), and low (0.3 m row spacing). Scale bar = 10 cm.

**Supplemental Figure S2. Map-based cloning of the gene associated with the *prose1* mutation.**

**(A, B)** Single nucleotide polymorphisms (SNPs) identified on chromosome 4A (Chinese Spring IWGSC RefSeq v1.0) through bulked segregant RNA-seq (BSR-seq) analysis. SNPs shown have an allele frequency difference (AFD) value greater than 0.8.

**Supplementary Figure S3. Sequence alignment of *Rht-A1a* and *Rht-A1h*.**

(A) Alignment of the coding sequences (CDSs) of *Rht-A1a* and *Rht-A1h.* The red box indicates a guanine (G)-to-adenine (A) base substitution in the coding region of *Rht-A1h* compared to *Rht-A1a*.

(B) Alignment of partial amino acids of Rht-A1a and Rht-A1h surrounding the mutant side. The dashed red box marks the conserved DELLA motif (D-E-L-L-A), and the solid red box highlights the glycine (G)-to-glutamic acid (E) substitution in Rht-A1h corresponding to the nucleotide variation shown in (A).

**Supplementary Figure S4. Maximum tiller inclination angles of *Rht-A1h-OE* and wild-type Fielder at the jointing stage.**

Fielder control and *Rht-A1h-OE* plants at the jointing stage. Scale bar = 10 cm.

**Supplementary Figure S5. Phenotypic analysis of *Rht-A1a*-OE T_1_ transgenic lines and *Rht-B1* expression in *Rht-B1a*-OE and *Rht-B1b*-OE lines.**

**(A)** Plant architecture of wild-type Fielder and *Rht-A1a*-OE at the jointing stage. Scale bar = 10 cm.

**(B)** Quantitative comparisons of tiller angle between Fielder and *Rht-A1a*-OE plants at the jointing stage. Data are mean ± SD; n = 15 individual plants.

**(C)** qRT-PCR analysis of *Rht-B1* expression in *Rht-B1a*-OE and *Rht-B1b*-OE lines (tiller bases). (Data are mean ± SD. Significant differences were determined by one-way ANOVA with Tukey’s HSD post hoc test (*P* < 0.05). Different lowercase letters denote statistically significant differences between groups. n = 3 biological replicates).

**Supplementary Figure S6.** **Overexpression of *Rht-B1b*, but not *Rht-B1a*, impairs shoot gravitropism in wheat.**

**(A)** Shoot curvature of Fielder, *Rht-B1a*-OE and *Rht-B1b*-OE seedlings after 48 h of gravistimulation under light conditions. Scale bar = 2 cm.

**(B)** Kinetics of shoot curvature under light conditions.

**(C)** Shoot curvature of Fielder, *Rht-B1a*-OE and *Rht-B1b*-OE seedlings after 48 h of gravistimulation under dark conditions. Scale bar = 2 cm.

**(D)** Kinetics of shoot curvature under dark conditions.

Data in (B) and (D) represent mean ± SD; n = 30 individual plants.

**Supplementary Figure S7.** **qRT-PCR analysis of *Rht-A1*, *Rht-B1*, and *Rht-D1* expression and *RUBY* reporter assay showing *Rht-A1* and *Rht-B1* spatial expression patterns.**

**(A)** Expression levels of *Rht-A1*, *Rht-B1* and *Rht-D1* in flag leaves and different internodes (INL1–INL5, from top to bottom) as determined by quantitative real-time PCR (qRT-PCR). Data are presented as mean ± SD (n = 3 biological replicates).

**(B)** *Rht-A1h _pro_::RUBY* and *Rht-B1_pro_::RUBY* expression in stem base and tiller buds. Scale bar = 0.5 cm.

**(C-F)** Tissue-specific promoter activity shown by RUBY staining: stem (C), spike (D, E), and seeds (F). Scale bars: 30 cm (C), 10 cm (D), 5 cm (E), 2 cm (F).

**Supplementary Figure S8. Comparison of interaction strengths between Rht-A1a/Rht-A1h and tiller angle regulators, and LCI analysis of Rht-B1a/B1b interaction with TaPROG1.**

**(A, B)** Quantitative LCI assays revealed that Rht-A1h exhibited a stronger interaction with TaLAZY1, TaLAZY4, and TaD14, but a weaker interaction with TaHST1L, in comparison with Rht-A1a.

**(C)** Quantitative LCI assays revealed that Rht-B1b exhibited a stronger interaction with TaPROG1 in comparison with Rht-B1a.

For **(A)** to **(C)**, Data are mean ± SD; ***P* < 0.01 (Student’s *t*-test).

**Supplementary Figure S9. AlphaFold-based structural predictions of Rht-A1h and Rht-A1a.**

**(A)** Superimposition of AlphaFold-predicted structures of Rht-A1a (blue) and Rht-A1h (red), showing the location of the G44E substitution adjacent to the DELLA motif. The N-terminal region exhibits conformational changes, while the GRAS domain remains largely unchanged.

**(B)** Residue distance plot calculated from structural alignment of the GRAS domains between Rht-A1a and Rht-A1h. The minimal residue distances throughout the GRAS domain indicate highly similar structures with no substantial conformational differences, whereas the N-terminal region (residues 1-160) shows pronounced distance variations.

**Supplementary Figure S10. Phenotypic analysis of *Rht-A1a-OE*, *Rht-A1h-OE*, and *TaPROG1-OE* transgenic wheat lines.**

**(A)** At the jointing stage, *TaPROG1-OE* lines exhibited significantly increased tiller angles. Scale bar = 4 cm.

**(B)** Phenotypic comparison of wild-type Fielder, *Rht-A1a-OE*#1, *Rht-A1h-OE*#1, and *TaPROG1-OE*#21 transgenic lines at the jointing stage. *Rht-A1a-OE*#1 plants showed tiller angles comparable to wild-type Fielder, whereas *Rht-A1h-OE*#1 and *TaPROG1-OE*#21 lines exhibited significantly enlarged tiller angles. And quantitative comparisons of tiller angle between Fielder, *Rht-A1a-OE*#1, *Rht-A1h-OE*#1, and *TaPROG1-OE*#21 plants at the jointing stage. Scale bar = 10 cm. Data are mean ± SD; n = 15 individual plants.

**Supplementary Figure S11. Prediction and validation of TaPROG1 binding to *TaWOX* promoters.**

**(A)** The figure shows multiple high-confidence TaPROG1-binding motifs identified in the promoters of *TaWOX6-A/B/D* and *TaWOX11-A/B/D* homoeologs.

**(B)** qRT-PCR validation confirming the expression levels of *TaWOX11-A/B/D* in *prose1*. Data are mean ± SD, ***P* < 0.01 (n = 3; Student’s *t*-test).

**(C)** qRT-PCR validation confirming the expression levels of *TaWOX11-A*, *-B*, and *-D* in *Rht-A1h-OE* (line #1) and *TaPROG1-OE* (line #21). Data are mean ± SD, ***P* < 0.01 (n = 3; Student’s *t*-test).

**(D)** Dual-luciferase assay showing that TaPROG1 and Rht-B1b independently suppress *TaWOX11-A* promoter activity, with additive effects when TaPROG1 and Rht-B1b co-expressed. Data are mean ± SD (n = 3; Student’s *t*-test).

**(E)** EMSA analysis confirms the binding of TaPROG1 to the *TaWOX11-B* promoter region. Competition assays with unlabeled probes show dose-dependent reduction of TaPROG1-DNA complex formation, demonstrating binding specificity.

**(F)** EMSA analysis confirms the binding of TaPROG1 to the *TaWOX11-D* promoter region. Competition assays with unlabeled probes show dose-dependent reduction of TaPROG1-DNA complex formation, demonstrating binding specificity.

**Supplementary Figure S12. CRISPR/Cas9-mediated mutagenesis of *TaWOX11* homoeologs in wheat.**

Schematic representation of the editing strategy and the resulting mutations in the *TaWOX11-A*, *TaWOX11-B* and *TaWOX11-D* genomic loci. Yellow highlighting indicates the CRISPR/Cas9 target sequences. PAM, protospacer adjacent motif.

**Supplementary Figure S13. Phenotypic analysis of *TaWOX11* mutants.**

**(A, B)** Fielder control and *Tawox11* (*Tawox11-aabbdd*) mutant plants at the anthesis stage. Scale bar = 10 cm.

**Supplementary Figure S14. Both *Rht-A1h* overexpression and *TaWOX11* mutation partially attenuate shoot gravity-sensing response in wheat.**

Shoot curvature of Fielder, *Rht-A1h-OE*, and *Tawox11* seedlings after 48 hours of gravistimulation in darkness. Scale bar = 2 cm.

**Supplementary Figure S15. Shoot gravitropism analysis of *Rht-A1a-OE* T_1_ seedlings compared to wild-type Fielder.**

Shoot curvature of Fielder and *Rht-A1a-OE*#1 seedlings after 48 h of gravistimulation under light/dark conditions and kinetics of shoot curvature under light/dark conditions. Scale bar = 2 cm. Data are mean ± SD; n = 30 individual plants.

**Supplementary Figure S16. *TaPROG1* negatively regulates shoot gravitropism partially through *TaWOX11* under light conditions.**

**(A)** Shoot curvature of Fielder, *TaPROG1-Flag*-Fielder, *Tawox11* and *TaPROG1-Flag*--*Tawox11* seedlings after 48 h of gravistimulation under light conditions. Scale bar = 2 cm.

**(B, C)** Kinetics of shoot curvature under light conditions. Data are mean ± SD; n = 30 individual plants. For (C), Significant differences were determined by one-way ANOVA with Tukey’s HSD post hoc test (*P* < 0.05). Different lowercase letters denote statistically significant differences between groups.

**Supplementary Figure S17. *TaPROG1* negatively regulates shoot gravitropism partially through *TaWOX11* under dark conditions.**

**(A)** Shoot curvature of Fielder, *TaPROG1-Flag*-Fielder, *Tawox11* and *TaPROG1-Flag*--*Tawox11* seedlings after 48 h of gravistimulation under dark conditions. Scale bar = 2 cm.

**(B)** Kinetics of shoot curvature under dark conditions. Data are mean ± SD; n = 30 individual plants.

**(C)** Western blot analysis of total protein extracts from Fielder, *Tawox11*, and their corresponding *TaPROG1-Flag* transient transgenic seedlings, using Anti-Flag antibody. β-Actin served as the loading control.

**Supplementary Figure S18. A working model contrasting wild-type *Rht-A1a* and mutant *Rht-A1h* in regulating wheat plant height and tiller angle.**

**(A)** Wild-type (*Rht-A1a*). In wild-type plants, Rht-A1a interacts with TaPROG1 and other partners with basal affinity, leading to normal repression of downstream target *TaWOX11*. This results in normal plant architecture characterized by upright tillers and standard plant height.

**(B)** Mutant (*Rht-A1h*). In contrast, this study identifies a novel semi-dominant *Rht-A1h* allele–generated via EMS mutagenesis. In *Rht-A1h* mutant plants, a single amino acid substitution (Gly-to-Glu) enhances the interaction strength between Rht-A1h and its partners (including TaPROG1, TaLAZY1, TaLAZY4, and TaD14), as indicated by the “Enhanced interaction” label and upward arrows (↑↑). This enhanced interaction leads to reinforced repression of *TaWOX11* expression, resulting in semi-dwarfism and a significant prostrate architecture with enlarged tiller angle and reduced plant height.
